# Causal relationship between gut microbiota and ankylosing spondylitis and potential mediating role of inflammatory cytokines: A mendelian randomization study

**DOI:** 10.1371/journal.pone.0306792

**Published:** 2024-07-31

**Authors:** Xinyu Du, Haibo Li, Hongzhou Zhao, Shuangshuang Cui, Xiaozhuo Sun, Xiaochan Tan

**Affiliations:** 1 Orthopedics and Traumatology Department of Integrated Traditional Chinese and Western Medicine, Tianjin Hospital, Tianjin, China; 2 Orthopedics Institute, Tianjin Hospital, Tianjin University, Tianjin, China; 3 Preventive Treatment of Disease Department, Second Affiliated Hospital of Tianjin University of Traditional Chinese Medicine, Tianjin, China; 4 Acupuncture Department, The First Affiliated Hospital of Zhejiang Chinese Medical University (Zhejiang Provincial Hospital of Traditional Chinese Medicine), Hangzhou, China; Khalifa University, UNITED ARAB EMIRATES

## Abstract

Associations between gut microbiota and ankylosing spondylitis have been discovered in previous studies, but whether these associations reflect a causal relationship remains inconclusive. Aiming to reveal the bidirectional causal associations between gut microbiota and ankylosing spondylitis, we utilized publicly available genome wide association study summary data for 211 gut microbiota (GM) taxa and ankylosing spondylitis (AS) to conduct two sample mendelian randomization analyses. Mediation analysis was performed to explore mediating inflammatory cytokines. We found that genetically predicted higher abundance of *Lactobacillaceae family*, *Rikenellaceae family* and *Howardella genus* had suggestive associations with decreased risk of ankylosing spondylitis while genetic proxied higher abundance of *Actinobacteria class* and *Ruminococcaceae_NK4A214_group genus* was associated with increased risk of ankylosing spondylitis. IL23 and IFN-γ were potential mediating cytokines for GM dysbiosis, especially for *Actinobacteria class*, leading to AS. Our study provided a new exploration direction for the treatment of AS. *Lactobacillaceae family*, *Rikenellaceae family*, *Howardella genus*, *Actinobacteria class* and *Ruminococcaceae_NK4A214_group genus* are expected to become new therapeutic targets and monitoring indicators for AS.

## Introduction

Ankylosing spondylitis (AS) is a chronic, progressive immune-mediated inflammatory disease with high heritability and disability, which particularly affects sacroiliac joints, paraspinal soft tissue, spinal process, and peripheral joints characterized by chronic pain, stiffness and functional limitation, consequently imposes a considerable burden on patients and society [1, 2]. The average number of AS cases per 10,000 was 23.8 in Europe, 16.7 in Asia, 31.9 in North America, 10.2 in Latin America and 7.4 in Africa [3]. The etiological mechanism of AS is complex and multifactorial involving genetics, infection and immunity, etc. [4, 5]. Treatments for AS mainly contain drug therapy, physical therapy, functional exercise and surgery, etc. Medications for AS mainly include non-steroidal anti-inflammatory drugs, disease modifying antirheumatic drugs, anti-tumor necrosis factor inhibitor and glucocorticoids, which showed a risk of gastrointestinal and cardiovascular side effects and potential infection [6].

Gut microbiota (GM) is an important part of human gastrointestinal system and exists in the form of co-benefits with human body [7]. GM is crucial for the maintenance of human health including participating in the development of mucosal immune system, maintaining metabolic stability, resistance to infection and production of certain neurotransmitters since birth [8]. The diversity and balance of GM can be disturbed by multiple endogenous and exogenous factors, which may lead to various diseases. Current studies have shown that the imbalance of GM is involved in the incidence and progression of many immune-related diseases, such as AS, inflammatory bowel diseases (IBD), reactive arthritis (RA), etc. [9–11].

Previous studies have investigated the relationship between GM and AS. Zhou et al. found that *Bacteroides coprophilus*, *Parabacteroides distasonis*, *Eubacterium siraeum*, *Acidaminococcus fermentans*, *Prevotella copri* were enriched in AS patients, while Enterococcus faecium E980 and TX0133a01 were reduced [12]. 16S rRNA sequencing analysis demonstrated a significant increase in the abundance of *Cyanobacteria*, *Deinococcota*, *Patescibacteria*, *Actinobacteriota*, *Synergistota* at phyla level and a relative declined amount of *Acidobacteriota*, *Bdellovibrionota*, *Campylobacterota*, *Chloroflexi*, *Gemmatimonadota*, *Myxococcota*, *Nitrospirota*, *Proteobacteria*, *Verrucomicrobiota* in AS patients [13]. Huang R et al. discovered that *Flavonifractor plautii*, *Oscillibacter*, *Parabacteroides distasonis*, *Bacteroides nordii* were increased and *Collinsella aerofaciens* was decreased in AS patients [14]. These studies have shown that there may be a relationship between GM and AS, but whether this relationship is causal has not been clearly elucidated due to the limitations of observational studies, such as confounding factors (e. g. lifestyle, drugs, diets), reverse causality, sample sizes, etc. Hence, it is imperative to clarify the causal relationship between GM and AS to provide an alternative for the prevention and treatment of AS by targeting the specific GM taxa.

Mendelian randomization (MR) is an increasingly used approach which can integrate data from genome-wide association studies (GWAS) and utilizes genetic variations as instrumental variables (IVs) to assess causal links between exposures and outcomes [15, 16]. It is highlighted that the variation stems from genetic factors rather than acquired changes affecting gene expression. For two-sample mendelian randomization(2SMR) analysis, SNPs robustly associated with exposure are selected as genetic IVs to infer causality by calculating the relative effect of the IVs-outcome association and the IVs-exposure association. Since genetic variants are allocated randomly during meiosis, MR results are unlikely to be influenced by environment that might confound the estimates. Therefore, the MR analysis is not subject to traditional confounding factors and reverse causation, making it easier to obtain true causality.

In this present study, we performed a bidirectional MR analysis and a mediation MR analysis to elucidate causal relationship between GM and AS basing on extensive GWAS data, and assumed potential mediating role of inflammatory cytokines. *Lactobacillaceae family*, *Rikenellaceae family*, *Howardella genus*, *Actinobacteria class and Ruminococcaceae_NK4A214_group genus* were identified as potential influencing factors for AS that may be targeted as potential targets for clinical or dietary interventions to improve the clinical performance of patients. Inflammatory cytokines (IL23 and IFN-γ) were found to play a potential role from GM dysbiosis to AS, especially for *Actinobacteria class*.

## Materials and methods

### Ethics statement

All GWAS summary data for GM and AS were collected from publicly published studies, and ethical reviews were approved by their respective study institutions. For our study, no additional basic data was required and all analyses were conducted online, thus no new ethical approval was required.

### Study design

**Fig 1:**
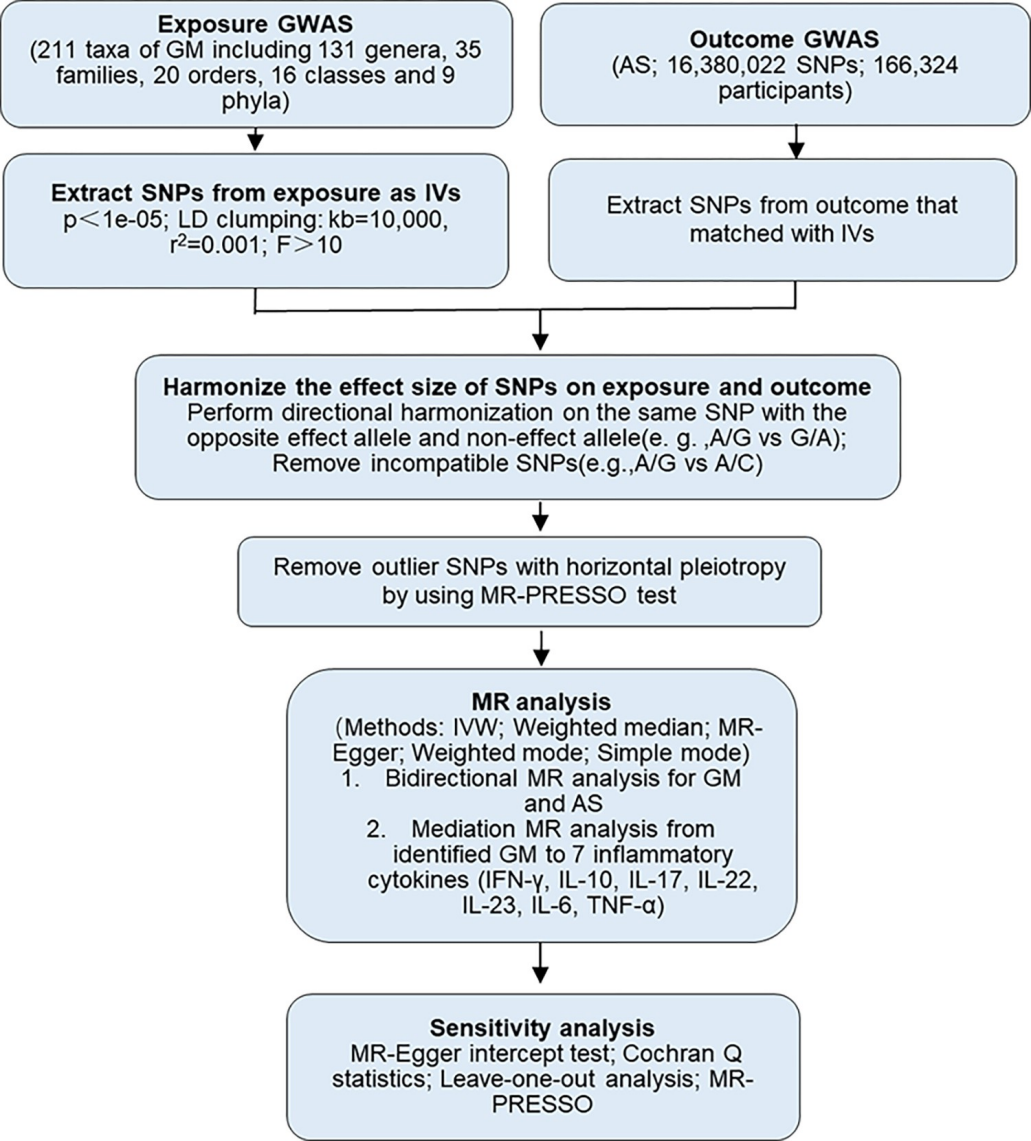
The flowchart of MR analyses between AS and GM. GWAS, genome wide association study; GM, gut microbiota; AS, ankylosing spondylitis; IVs, instrumental variables; SNPs, single nucleotide polymorphisms; MR, mendelian randomization; IVW, inverse variance weighted; MR-PRESSO, mendelian randomization-pleiotropy residual sum and outlier.

**Fig 2:**
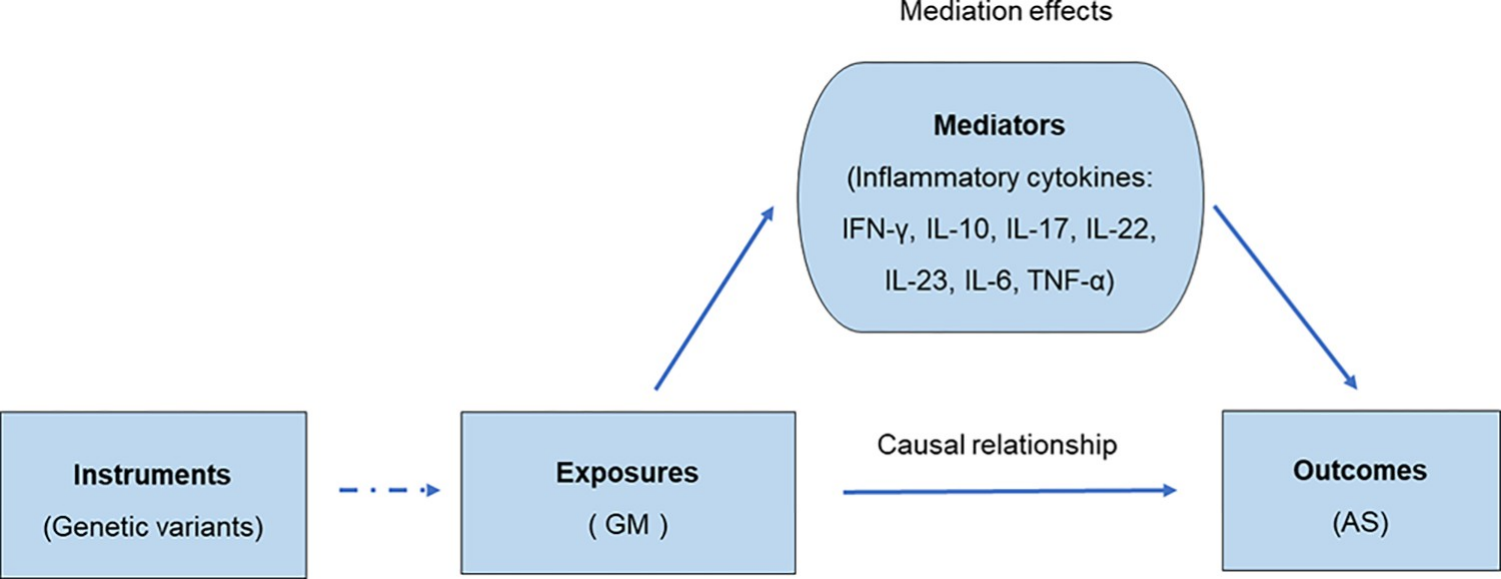
Diagrams illustrating associations for mediation MR analysis.

The flowchart briefly described our study was shown in Fig 1. The MR analysis relies on three basic assumptions: i) IVs are strongly associated with exposure; ii) IVs are independent of confounding factors associated with exposure and outcome; iii) IVs are associated with outcome only through exposure (Fig 2) [17].

In this study, we utilized GWAS database concerning about 211 GM taxa and AS. A series of quality control steps were taken to select instrumental single nucleotide polymorphisms (SNPs) that were strongly associated with GM as instrumental variables (IVs). Inverse-variance weighted (IVW), weighted median and MR-Egger were performed to estimate the causal association from each GM taxa to the risk of AS followed by sensitivity analysis to assess the robustness of MR results. Next, we conducted a reverse MR analysis to explore the effect of AS on GM. Outlier SNPs were identified by mendelian randomization-pleiotropy residual sum and outlier (MR-PRESSO) test and removed. 7 inflammatory cytokines, including IFN-γ [18, 19], IL-10 [20, 21], IL-17 [22, 23], IL-22 [22, 23], IL-23 [24, 25], IL-6 [26, 27] and TNF-α [20, 28, 29], which have been widely reported to be involved in the pathogenesis of AS, were then used as mediate variants, and a mediation MR analysis was performed, that is, the identified GM taxa were chosen as the exposure, and the inflammatory cytokines were the outcome. Sensitivity analyses including MR-Egger intercept test, Cochran’s Q test and leave-one-out analysis were performed to validate the accuracy and robustness of the results.

### GWAS summary data

To make our results more accurate and comprehensive, we selected large-scale GWAS summary data of GM and AS. A total of 211 GM taxa were analyzed, including 131 genera, 35 families, 20 orders, 16 classes and 9 phyla. GWAS summary data for GM could be acquired online (*https*:*//mibiogen*.*gcc*.*rug*.*nl/*) based on the research of Kurilshikov A et al. In brief, genome-wide genotypes and 16S fecal microbiome data in 18, 340 individuals from 24 cohorts were analyzed in this research. Relevant systems and software were applied for genetic data processing. Spearman’s correlation analysis was used to identify the associations between genetic loci and the abundance of GM after adjustment for age, sex, technical covariates and genetic principal components [30].

GWAS summary data of AS was extracted directly from IEU GWAS database(*https*:*//gwas*.*mrcieu*.*ac*.*uk/*, dateset ID = *finn-b-M13_ANKYLOSPON*) comprising 166,324 European participants and 16,380,022 SNPs. Here, the disease classification of AS is “XIII Diseases of the musculoskeletal system and connective tissue (M13_)”, and is defined as “an autoimmune chronic inflammatory disease characterized by inflammation of the vertebral and sacroiliac joints of the spine, with patients presenting with spinal stiffness and pain.”

GWAS summary data of inflammatory cytokines were also obtained from IEU GWAS database, and the basic information was shown in Table 1.

**Table 1:** GWAS data information for inflammatory cytokines.

Cytokine	PMID	Sample size	nSNPs	Author
IFN-γ/IL-10/ IL-22/IL-23	29875488	3,301	10,534,735	Sun BB [31]
IL-6	33303764	1,301	18,166,693	Gilly A [32]
TNF-α	27989323	3,475	9,512,914	Ahola-Olli AV [33]
IL-17	27989323	7,760	9,786,653	Ahola-Olli AV [33]

IL, interleukin; IFN, interferon; TNF, tumor necrosis factor

### Selection of robust genetic IVs and extraction of outcome-SNPs

To obtain robust genetic IVs from exposure, strict inclusion and exclusion criteria were set. A significance cut-off value of P <5×10^−8^ was first employed to screen out SNPs that were statistically associated with exposure. If very few SNPs were extracted from exposure, an appropriately eased threshold (P <1×10^−5^) was chosen to include more SNPs as IVs [34]. SNPs with linkage disequilibrium (LD) were removed by stringent clumping criteria (kb = 10,000, r^2^ = 0.001) to retain mutual independent instrumental SNPs. F-statistic was calculated by the formula (F = β^2^/SE^2^) to appraise the potency of each SNP [35, 36]. β stands for effect size, SE stands for standard error. An SNP with F-statistic value less than 10 was identified as a weak genetic IV and eliminated. Then the outcome-SNPs were extracted from GWAS summary data of outcome matched with IVs.

To estimate whether the IVs were related to the potential confounding factors such as age, gender and HLA-27 which are correlated with AS, phenoscanner platform (*www*.*Phenoscanner*.*medschl*.*cam*.*ac*.*uk*) was utilized which exhibited comprehensive data of the association between genotype and phenotype. IVs associated with potential confounders were further excluded to ensure the accuracy of MR analyses.

### Harmonization for the effect size of SNPs on exposure and outcome

Directional harmonization was performed on the same SNP with the opposite effect allele and non-effect allele in exposure and outcome (e.g., the effect allele and non-effect allele of one SNP were A/G in exposure and G/A in outcome) to ensure the effect of the SNP on exposure and outcome were corresponded to the same allele. Moreover, incompatible SNPs (e.g., the effect allele and non-effect allele of one SNP were A/G in exposure and A/C in outcome) were removed to ensure the obtained SNPs were all valid.

### MR analyses

Based on the above-retained SNPs, MR analyses were performed utilizing three MR methods, namely, IVW, weighted median and MR-Egger, to estimate causal effects. Causal effects were calculated as the SNP-outcome effect estimate divided by the SNP-exposure effect estimate. Each statistical method has its own assumption model as follows [34, 37–40]. The IVW method assumes all SNPs are valid IVs and no horizontal pleiotropy exist. The weighted median method assumes over half of IVs are valid. MR-Egger regression can provide consistent estimates when 100% of genetic variants are invalid IVs and relies on the Instrument Strength Independent of Direct Effect (InSIDE) condition that instrument-exposure and pleiotropic effects are uncorrelated. The weighted model method can obtain a robust estimate when a plurality of genetic variants are valid IVs. The simple mode can provide robustness for pleiotropy. IVW method, which can provide an optimally efficient estimate when all included SNPs can be used as effective IVs [40]. Therefore, if there is no heterogeneity or pleiotropy, our results were mainly based on the IVW method and supplemented by weighted median, MR-Egger, weighted mode, and simple mode methods. If heterogeneity exists in our MR analysis, we will continue to conduct MR-PRESSO test and exclude unqualified SNPs until the P-value of the heterogeneity test is greater than 0.05. The plus or minus of beta value represents a positive or negative correlation between exposure and outcome. Only when the P-value of the IVW method is less than statistical threshold and the beta direction (plus or minus) of the IVW method is consistent with the other methods in the meantime, we will agree that there is a statistically significant causal relationship between exposure and outcome.

### Reverse-direction MR analysis

To avoid reverse causality interfering with the results of our study, the direction of causation was further explored by reversing the exposure and outcome, which means AS was regarded as exposure and GM which had causal effects on AS was regarded as outcome.

### Sensitivity analysis

To validate the robustness and accuracy of our results, four methods of sensitivity analysis were implemented, namely, MR-Egger intercept test, Cochran’s Q test, leave-one-out analysis and MR-PRESSO test. The intercept term in MR-Egger can provide an estimate of horizontal pleiotropy. Cochran’s Q test was used to quantify heterogeneity. Leave-one-out analysis was used to identify whether the result was driven by a single SNP through leaving out each SNP in turn. MR-PRESSO test can also provide a horizontal pleiotropy detection.

All results were expressed as beta, p-value, odds ratio (OR) and corresponding 95% confidence interval (95%CI). Beta value can reflect the effect between exposure and outcome. When beta is positive, it indicates that increased exposure will lead to increased risk of outcome; when beta is negative, it indicates that increased exposure will reduce the risk of outcome. We applied Bonferroni correction aiming to obtain a more accurate assessment of the causal relationship. A p-value was considered statistically significant when less than 0.000237 (0.05/211) and was considered as a suggestive evidence of potential causal associations when between 0.000237 and 0.05. All analyses were performed with R software (version 4.2.2). IVW, weighted median, MR-Egger and leave-one-out analysis were performed with “Two Sample MR” package. MR-PRESSO test was performed with “MRPRESSO” package.

## Results

### Causal effects of GM on AS

**Fig 3:**
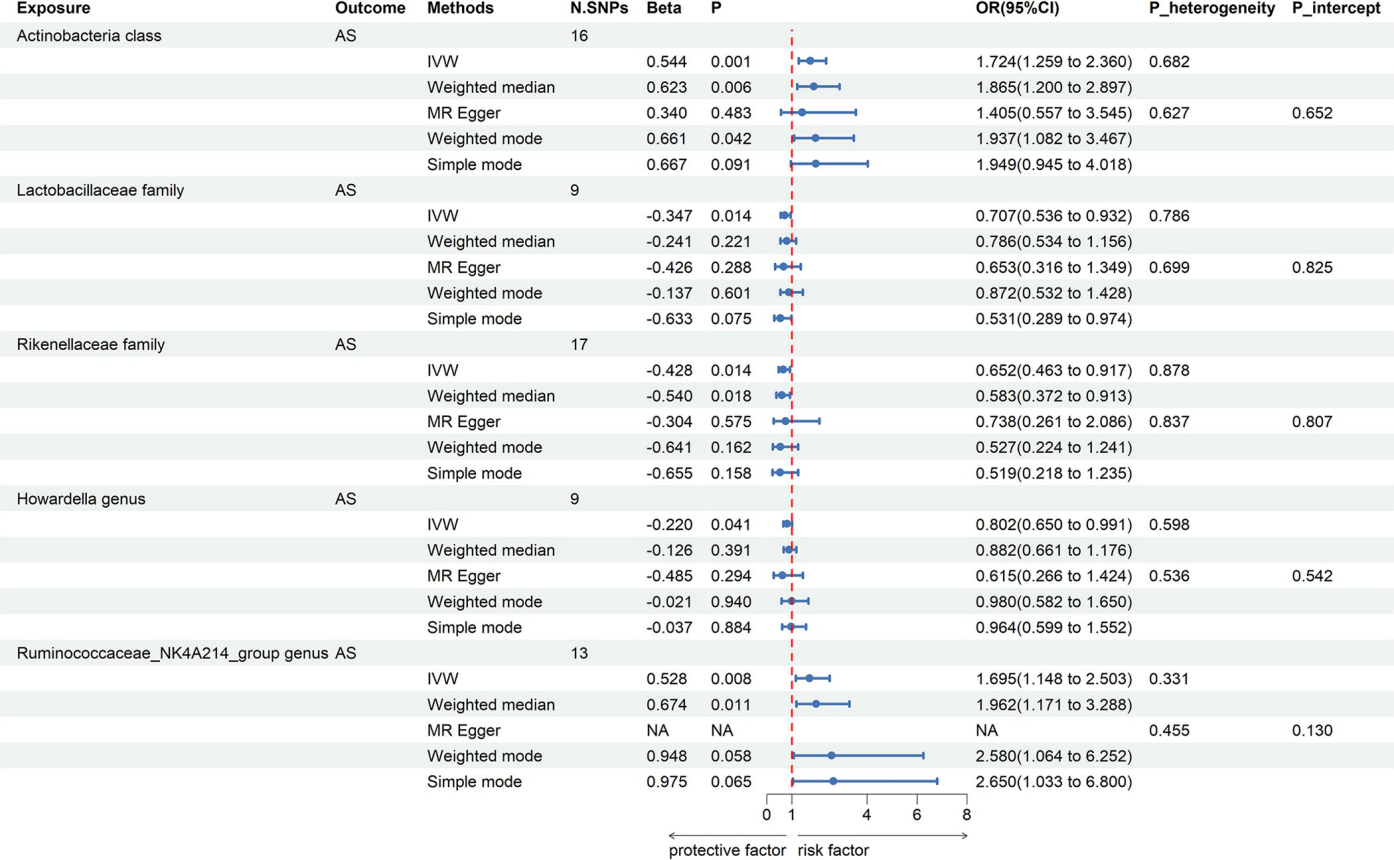
Suggestively causal effects of GM on AS. P_heterogeneity represents the p-value obtained by Cochran’s Q test. P_intercept indicates p-value obtained by MR-Egger intercept test. NA stands for anomaly estimate. The forest plot represents the pooled odds ratio and 95% confidence interval under IVW, weighted median, and MR-Egger methods. N. SNPs represents the final number of SNPs retained for MR analyses. AS, ankylosing spondylitis; IVW, inverse variance weighted; OR, odds ratio; CI, confidence interval.

**Fig 4:**
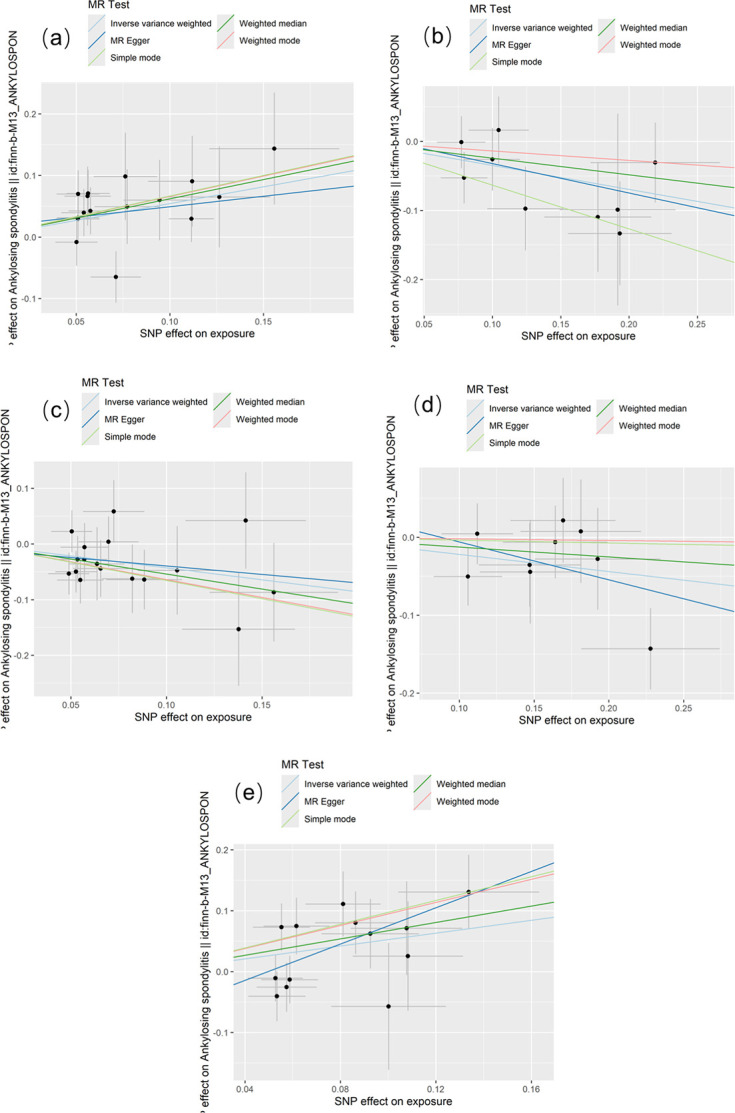
Scatter plots for suggestively causal effects of GM on AS. The 95%CI of odd ratio (OR) for each IV is presented by vertical and horizontal lines. The slope of the lines represents estimated causal effect of MR methods. Light blue lines represent estimations with IVW method. Blue lines represent estimations with MR-Egger method. Green lines represent estimations with weighted median method. Light green lines represent estimations with simple mode method. Red lines represent estimations with weighted mode method. (a): Scatter plot of 5 MR methods for causal effect of *Actinobacteria class* on AS. (b): Scatter plot of 5 MR methods for causal effect of *Lactobacillaceae family* on AS. (c): Scatter plot of 5 MR methods for causal effect of *Rikenellaceae family* on AS. (d): Scatter plot of 5 MR methods for causal effect of *Howardella genus* on AS. (e): Scatter plot of 5 MR methods for causal effect of *Ruminococcaceae_NK4A214_group genus* on AS. MR, mendelian randomization. GM, gut microbiota; AS, ankylosing spondylitis; SNPs, single nucleotide polymorphisms.

All IVs were qualified for F-statistics>10, indicating no evidence of weak instrument bias and our selected IVs were strongly correlated with exposure. The significant results of MR analyses were presented in Figs 3 and 4. Using the IVW estimate, genetically predicted higher abundance of *Actinobacteria class* was suggestively associated with higher risk of AS (Beta: 0.544, OR: 1.724, 95%CI: 1.259, 2.360, P = 0.001). The result predicted by the weighted median (Beta: 0.623, OR: 1.865, 95%CI: 1.200, 2.897, P = 0.006) and weighted mode (Beta: 0.661, OR: 1.937, 95%CI: 1.082, 3.467, P = 0.042) were compatible with the IVW estimate. The result of MR-Egger method (Beta: 0.340, OR: 1.405, 95%CI: 0.557, 3.545, P = 0.483) and simple mode (Beta: 0.667, OR: 1.949, 95%CI: 0.945, 4.018, P = 0.091) although failed to detect statistically significant associations but revealed the similar effect trend. Genetically predicted higher abundance of *Ruminococcaceae_NK4A214_group genus* was suggestively associated with higher risk of AS by IVW estimate (Beta: 0.528, OR: 1.695, 95%CI: 1.148, 2.503, P = 0.008). The results predicted by the weighted median (Beta: 0.674, OR: 1.962, 95%CI: 1.171, 3.288, P = 0.011) was compatible with the IVW estimate. The result of weighted mode method (Beta: 0.948, OR: 2.58, 95%CI: 1.064, 6.252, P = 0.058) and simple mode (Beta: 0.975, OR: 2.65, 95%CI: 1.033, 6.8, P = 0.065) although failed to detect statistically significant associations but revealed the similar effect trend. Moreover, according to IVW method, it was found that genetically predicted higher abundance of *Lactobacillaceae family* was suggestively in relation to lower risk of AS by IVW estimate (Beta: -0.347, OR: 0.707, 95%CI: 0.536, 0.932, P = 0.014). Similar changing trends were also detected in weighted median (Beta: -0.241, OR: 0.786, 95%CI: 0.534, 1.156, P = 0.221), MR-Egger (Beta: -0.426, OR: 0.653, 95%CI: 0.316, 1.349, P = 0.288), weighted mode (Beta: -0.137, OR: 0.872, 95%CI: 0.532, 1.428, P = 0.601) and simple mode (Beta: -0.633, OR: 0.531, 95%CI: 0.289, 0.974; P = 0.075) methods. Using the IVW estimate, genetically predicted higher abundance of *Rikenellaceae family* was suggestively in relation to lower risk of AS (Beta: -0.428, OR: 0.652, 95%CI: 0.463, 0.917, P = 0.014). The findings predicted by the weighted median method (Beta: -0.540, OR: 0.583, 95%CI: 0.372, 0.913, P = 0.018) was compatible with the IVW estimate. The MR-Egger (Beta: -0.304, OR: 0.738, 95%CI: 0.261, 2.086, P = 0.575), weighted mode (Beta: -0.641, OR: 0.527, 95%CI: 0.224, 1.241, P = 0.162) and simple mode (Beta: -0.655, OR: 0.519, 95%CI: 0.218, 1.235, P = 0.158) approaches although failed to detect statistically significant associations but revealed the similar effect trend. Genetically predicted higher abundance of *Howardella genus* was suggestively in relation to lower risk of AS by IVW estimate (Beta: -0.220, OR: 0.802, 95%CI: 0.650, 0.991, P = 0.041). Similar changing trends were also detected in weighted median (Beta: -0.126, OR: 0.882, 95%CI: 0.661, 1.176, P = 0.391), MR-Egger (Beta: -0.485, OR: 0.615, 95%CI: 0.266, 1.424, P = 0.294), weighted mode (Beta: -0.021, OR: 0.980, 95%CI: 0.582, 1.650, P = 0.940) and simple mode (Beta: -0.037, OR: 0.964, 95%CI: 0.599, 1.552, P = 0.884) methods.

**Fig 5:**
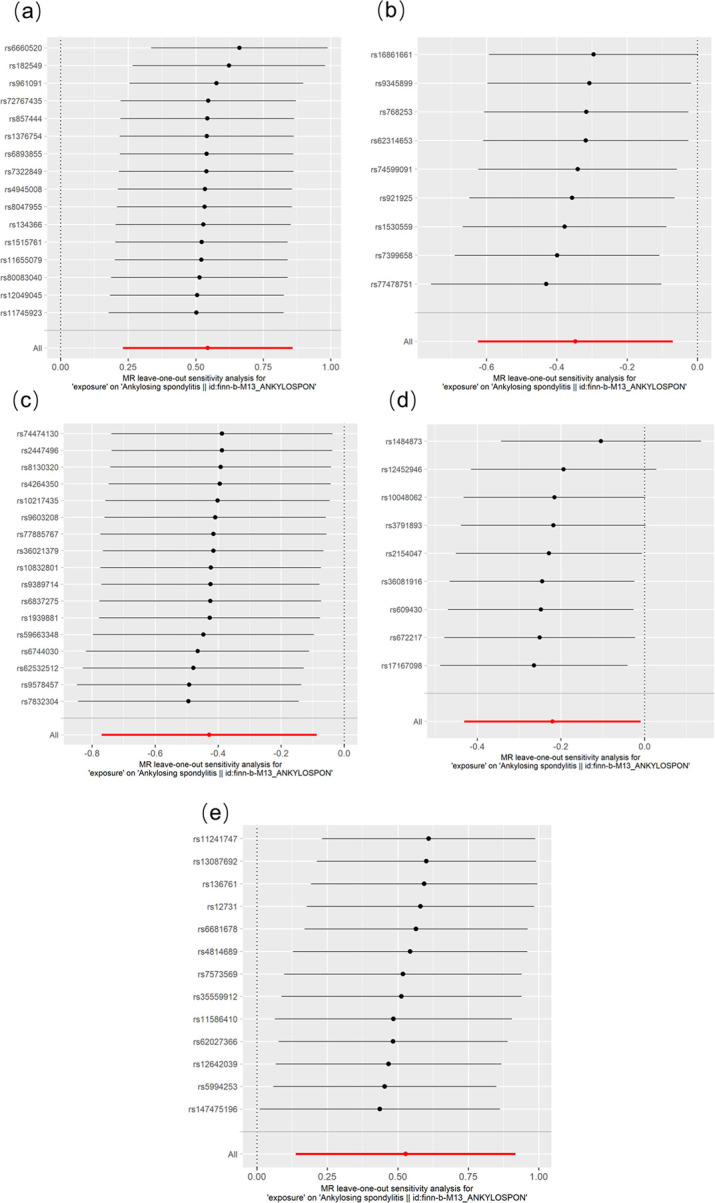
Results of leave-one-out analysis for suggestively causal effects of GM on AS. The figures showed the MR analysis results after phasing out single SNP one by one. Red lines represent estimations from IVW test. (a): Forest plots for *Actinobacteria class* on AS. (b): Forest plots for *Lactobacillaceae family* on AS. (c): Forest plots for *Rikenellaceae family* on AS. (d): Forest plots for causal effect of *Howardella genus* on AS. (e): Forest plots for causal effect of *Ruminococcaceae_NK4A214_group genus* on AS. AS, ankylosing spondylitis.

Sensitivity analyses showed no horizontal pleiotropy or significant heterogeneity (Fig 5), so our MR results was accurate and reliable. Detailed information on the IVs of 5 suggestive GM taxa was shown in S1 File. The final retained SNPs of 5 suggestive GM taxa and AS after directional harmonization were shown in S2 File.

### Causal effects of AS on GM

**Fig 6:**
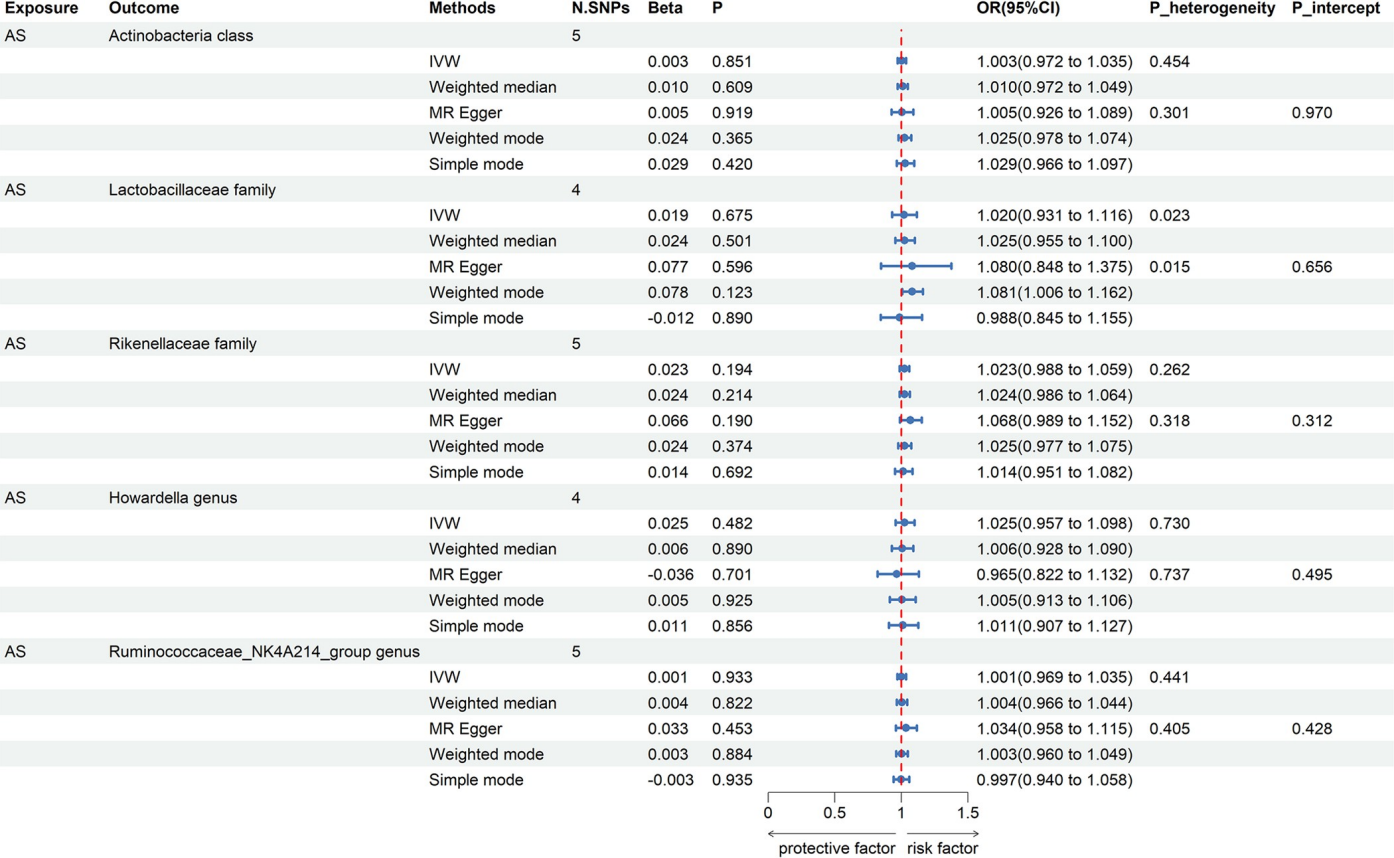
Causal effects of AS on GM. P_heterogeneity represents the p-value obtained by Cochran’s Q test. P_intercept indicates p-value obtained by MR-Egger intercept test. The forest plot represents the pooled odds ratio and 95% confidence interval under the IVW, Weighted median, and MR-Egger methods. N. SNPs represents the final number of SNPs retained for MR analyses. AS, ankylosing spondylitis; IVW, inverse variance weighted; OR, odds ratio; CI, confidence interval.

All IVs for AS were qualified for F-statistics >10, which indicated that our selected IVs did not have weak IVs bias and were all strongly correlated with exposure. Reverse-direction MR analyses showed no causal relationship of AS on GM (Fig 6). By using the IVW estimate, our study was unable to identify any causal associations from AS to *Actinobacteria class* (Beta: 0.003, OR: 1.003, 95%CI: 0.972, 1.035, P = 0.851), *Lactobacillaceae family* (Beta: 0.019, OR: 1.020, 95%CI: 0.931, 1.116, P = 0.675), *Rikenellaceae family* (Beta: 0.023, OR: 1.023, 95%CI: 0.988, 1.059, P = 0.194), *Howardella genus* (Beta: 0.025, OR: 1.025, 95%CI: 0.957, 1.098, P = 0.482) and *Ruminococcaceae_NK4A214_group genus* (Beta: 0.001, OR: 1.001, 95%CI: 0.969, 1.035, P = 0.933). Similarly, the predicted findings of weighted median method for causal associations from AS to Actinobacteria class (Beta: 0.010, OR:1.010, 95%CI: 0.972, 1.049, P = 0.609), *Lactobacillaceae family* (Beta: 0.024, OR: 1.025, 95%CI: 0.955, 1.100, P = 0.501), *Rikenellaceae family* (Beta: 0.024, OR: 1.024, 95%CI: 0.986, 1.064, P = 0.214), *Howardella genus* (Beta: 0.006, OR: 1.006, 95%CI: 0.928, 1.090, P = 0.890) and *Ruminococcaceae_NK4A214_group genus* (Beta: 0.004, OR: 1.004, 95%CI: 0.966, 1.044, P = 0.822) were compatible with the IVW estimate. MR-Egger approach also yielded consistent estimates with the other two methods for the causal associations from *Actinobacteria class* (Beta: 0.005, OR: 1.005, 95%CI: 0.926, 1.089, P = 0.919), *Lactobacillaceae family* (Beta: 0.077, OR: 1.080, 95%CI: 0.848, 1.375, P = 0.596), *Rikenellaceae family* (Beta: 0.066, OR: 1.068, 95%CI: 0.989, 1.152, P = 0.190), *Howardella genus* (Beta: -0.036, OR: 0.965, 95%CI: 0.822, 1.132, P = 0.701), *Ruminococcaceae_NK4A214_group genus* (Beta: 0.033, OR: 1.034, 95%CI: 0.958, 1.115, P = 0.453) to AS. The predicted findings of weighted mode method for causal associations from AS to *Actinobacteria class* (Beta: 0.024, OR: 1.025, 95%CI: 0.978, 1.074, P = 0.365), *Lactobacillaceae family* (Beta: 0.078, OR: 1.081, 95%CI: 1.006, 1.162, P = 0.123), *Rikenellaceae family* (Beta: 0.024, OR: 1.025, 95%CI: 0.977, 1.075, P = 0.374), *Howardella genus* (Beta: 0.005, OR: 1.005, 95%CI: 0.913, 1.106, P = 0.925) and *Ruminococcaceae_NK4A214_group genus* (Beta: 0.003, OR: 1.003, 95%CI: 0.960, 1.049, P = 0.884) were compatible with the IVW estimate. Simple mode approach also yielded consistent estimates with IVW method for the causal associations from Actinobacteria class (Beta: 0.029, OR: 1.029, 95%CI: 0.966, 1.097, P = 0.420), *Lactobacillaceae family* (Beta: -0.012, OR: 0.988, 95%CI: 0.845, 1.155, P = 0.890), *Rikenellaceae family* (Beta: 0.014, OR: 1.014, 95%CI: 0.951, 1.082, P = 0.692), *Howardella genus* (Beta: 0.011, OR: 1.011, 95%CI: 0.907, 1.127, P = 0.856), *Ruminococcaceae_NK4A214_group genus* (Beta: -0.003, OR: 0.997, 95%CI: 0.940, 1.058, P = 0.935) to AS. Sensitivity analyses showed no horizontal pleiotropy or significant heterogeneity, so our MR results were accurate and reliable. Detailed information on the IVs of AS was shown in S3 File. The final retained SNPs of AS and 5 suggestive GM taxa after directional harmonization were shown in S4 File.

### Mediating effects of inflammatory cytokines

Using the IVW estimate, genetically predicted higher abundance of Actinobacteria class was associated with increased levels of IL23 (Beta: 0.339, OR: 1.403, 95%CI: 1.124, 1.752, P = 0.003) and IFN-γ (Beta: 0.280, OR: 1.323, 95%CI: 1.060, 1.652, P = 0.013) (Table 2). The result predicted by the weighted median, MR-Egger, weighted mode and simple mode although failed to detect statistically significant associations but revealed the similar effect trend Sensitivity analyses showed no horizontal pleiotropy or significant heterogeneity, so our MR results were accurate and reliable. All MR results were presented in S5 File.

**Table 2:** Causal effects of Actinobacteria class on IL23 and IFN-γ.

Expoure	Outcome	Methods	N.SNPs	Beta	P	OR(95%CI)	P__heterogeneity_	P__intercept_
*Actinobacteria class*	IL23	IVW	15	0.339	0.003	1.403(1.124, 1.752)	0.552	
		Weighted median		0.151	0.340	1.163(0.853, 1.587)		
		MR-Egger		0.106	0.754	1.111(0.581, 2.125)	0.518	0.466
		Weighted mode		0.063	0.762	1.065(0.713, 1.591)		
		Simple mode		0.022	0.942	1.022(0.577, 1.810)		
	IFN-γ	IVW	15	0.280	0.013	1.323(1.060, 1.652)	0.757	
		Weighted median		0.290	0.074	1.337(0.972, 1.838)		
		MR-Egger		0.357	0.300	1.428(0.747, 2.731)	0.693	0.809
		Weighted mode		0.314	0.206	1.368(0.860, 2.176)		
		Simple mode		0.376	0.192	1.457(0.851, 2.495)		

P_heterogeneity represents the p-value obtained by Cochran’s Q test.

P_intercept indicates p-value obtained by MR-Egger intercept test.

N. SNPs represents the final number of SNPs retained for MR analyses.

IL, interleukin; IFN, interferon; IVW, inverse variance weighted; OR, odds ratio; CI, confidence interval.

## Discussion

Associations between GM and AS have been discovered in previous studies, but whether these associations reflect a causal relationship remains inconclusive. In our research, we conducted a comprehensive MR study using large-scale GWAS data for 211 GM taxa and AS, and performed a reverse causality analysis. We discovered that the abundance of Actinobacteria class and Ruminococcaceae_NK4A214_group genus predicted by genetics were positively correlated with AS, while the abundance of *Lactobacillaceae family*, *Rikenellaceae family* and *Howardella genus* predicted by genetics were negatively correlated with AS. Reverse-direction MR analyses showed no causal relationship from AS to above 5 significant GM. IL23 and IFN-γ were potential mediators for GM dysbiosis leading to AS, especially for Actinobacteria class.

Actinobacteria is known to produce a wide range of secondary metabolites and facilitate the development of AS through modulating the ubiquitination of IκB-α which may contribute to the activation of NF-κB signaling and the accumulation of proinflammatory factors in patients with AS [2, 41]. A case-control study have reported that fecal microbiota in AS patients was characterized by a higher abundance of Actinobacteria [7]. This phenomenon could be authenticated to some extent by our results that a higher abundance of *Actinobacteria class* was a risk factor for AS. QH Dai et al. observed dysfunction of the microbiota in ankylosing spondylitis patients, characterized by a reduced abundance of SCFA-producing bacteria [42]. SCFAs play an indispensable role in maintaining intestinal immune homeostasis through increasing the secretion of anti-inflammatory cytokines and attenuating HLA-B27-related inflammation [43]. Due to the ability to produce SCFAs, especially butyric acid, *Ruminococcaceae* is believed to have an anti-inflammatory effect, capable of inhibiting the activation of NF-κB. However, studies found that *Ruminococcaceae* is overexpressed in patients with sacroiliitis and associated with onset of AS [44]. IgA-SEQ analysis revealed an increased abundance of *Ruminococcaceae* in the stool of axial spondyloarthritis patients [45]. Our study further found that higher abundance of *Ruminococcaceae_NK4A214_group genus* was associated with increased risk of AS. The intrinsic mechanism of *Ruminococcaceae* on AS needs to be further studied in the future.

*Lactobacillaceae*, a type of lactic acid bacteria, is beneficial for maintaining intestinal health and often used as a prebiotic [46]. Although researches on the association of *Lactobacillaceae* and AS are currently limited, our comprehensive MR analyses showed the protective effect of *Lactobacillaceae family* on AS. We speculated that the internal mechanism may be related to its ability to produce short-chain fatty acids (SCFAs). *Rikenellac*eae has been found to be related to the expression of HLA-A24 and HLA-B27 [44, 47]. One study found that the increase of *Rikenellaceae* abundance was negatively correlated with the production of pro-inflammatory cytokines, which could explain our findings that *Rikenellaceae family* is a protective factor against AS [48]. *Howardella* has been rarely reported and it was shown that spinal muscular atrophy patients had a decreased *Howardella* abundance [49]. Our research showed that *Howardella genus* was negatively correlated with AS, however the association between *Howardella* and AS pathogenesis needs further exploration.

Some GM taxa such as *Lactobacillus*, *Bifidobacterium*, *Enterococcus* and *Streptococcus* have been used as prebiotics to treat AS clinically [9]. The mechanism mainly includes the production of bactericidal substances, competition with pathogens for intestinal epithelial binding sites, improving immunity through activation of toll-like receptors (TLRs), regulating inflammatory response and improving intestinal barrier function [50]. However, in a randomized controlled trial, probiotic therapy with three bacterial strains (*Streptococcus salivatus K12*, *Bifidobacterium lactis LAFTI B94*, *Lactobacillus acidophilus LAFTI L10*) showed no significant benefit in patients with active spondylarthritis after 12 weeks of treatment, as compared with placebo group. We speculate that this unsatisfactory effect may be related to the inaccurate selection of GM taxa as therapeutic targets. The authors also noted that there was very limited data to guide the selection of probiotic taxa [51]. Due to the unavoidable confounding factors in the existing studies, the confidence of the relationship between GM and AS derived from these studies is limited. Therefore, there is an urgent need to identify the exact GM taxa associated with AS to guide the clinical treatment. In this study, 5 GM taxa suggestively associated with AS were identified by MR analyses. We advised to further explore their feasibilities as therapeutic targets for AS. Besides, people with higher abundance of the intestinal *Actinobacteria class* and *Ruminococcaceae_NK4A214_group genus* or lower abundance of the intestinal *Lactobacillaceae family*, *Rikenellaceae family* and *Howardella genus* than normal should be considered at high risk for the AS disease. Blood HLA-B27 testing, spine and sacroiliac joint X-rays are recommended if they have a complain of back pain or hip pain.

**Fig 7:**
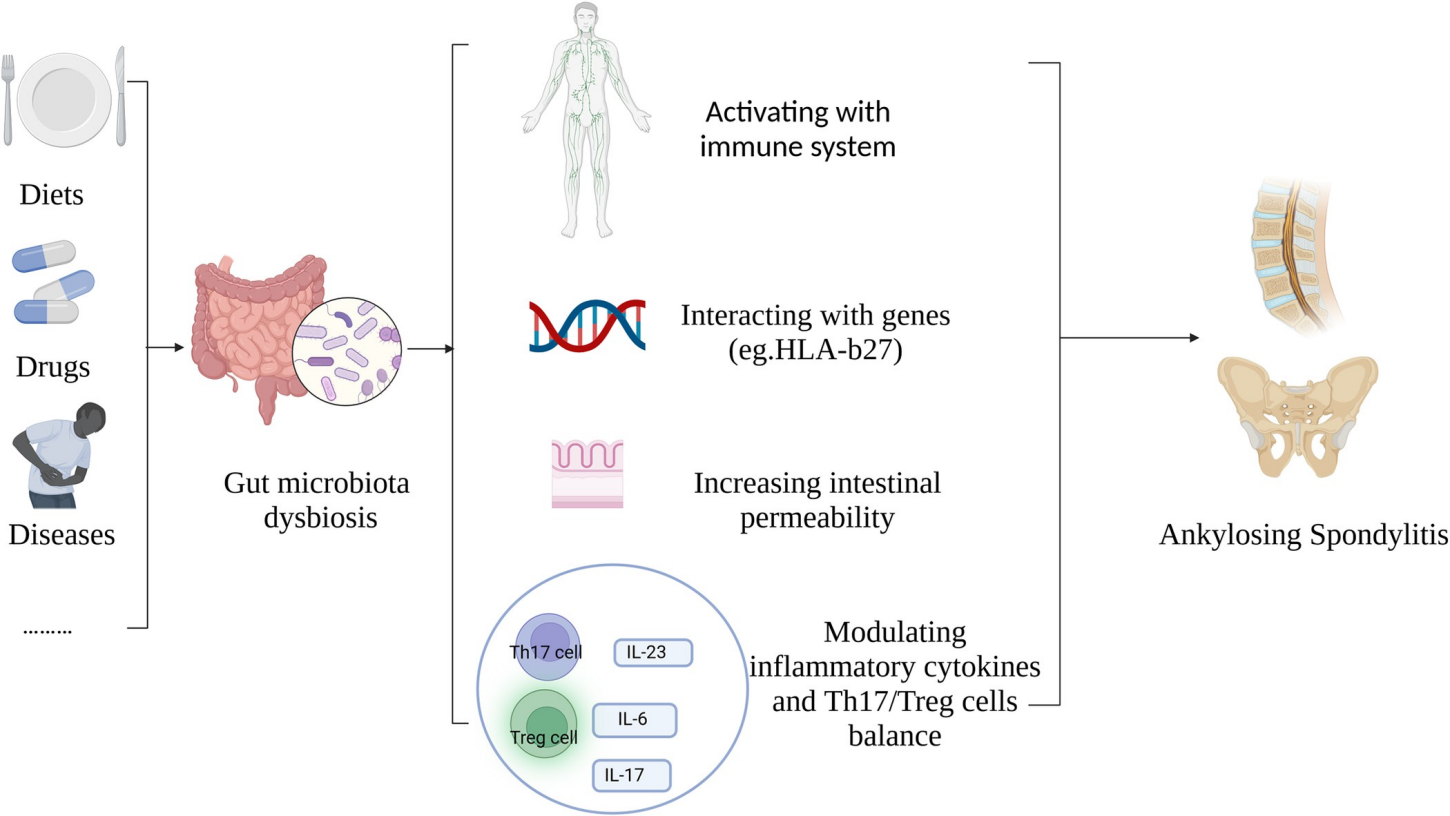
The internal relationship between GM and AS. GM, gut microbiota; AS, ankylosing spondylitis.

Overall, dysbiosis of GM is closely related to the pathogenesis and susceptibility for AS through various mechanism such as increasing intestinal permeability, activating the gut mucosa immune system, interacting with genes (e.g., HLA-B27), modulating inflammatory cytokines and Th17/Treg cells balance [52] (Fig 7). Inflammatory cytokines such as IFN-γ [18, 19], IL-10 [20, 21], IL-17 [22, 23], IL-22 [22, 23], IL-23 [24, 25], IL-6 [26, 27] and TNF-α [20, 28, 29], have been widely reported to be involved in the pathogenesis of AS. Particularly, the IL-23/IL-17 immune axis has been shown to be an important factor in the immunopathogenesis of AS. IL-23 and IL-17, the key cytokines in AS, are produced in the inflamed gut of AS and IBD [52]. Our study shows that *Actinobacteria class* can significantly promote the secretion of proinflammatory cytokine IL23 and IFN-γ, which were widely reported to promote the development of AS, indicating IL23 and IFN-γ probably played a mediating role from GM dysbiosis to AS. Active intestinal inflammation has been related to increased disease activity in AS [53]. In view of similarities in the aberrations of GM in IBD and AS [7], dysbiosis of GM might play an important role in the pathogenesis of both diseases. Of note, previous MR studies have shown IBD has a positive causal effect on AS [54], while GM has no causal effect on IBD [55], indicating that IBD may not be an intermediate mechanism of GM causing AS.

Nevertheless, the present study also had some limitations. First, previous studies indicated that the onset of AS is age and gender related [56], while we did not perform subgroup analysis by gender or age because of constrained and unattainable individual data. Second, GWAS summary data of exposure and outcome were obtained primarily from the European population, which may have skewed our findings by race and affected the extrapolation of our findings to general population. Third, the most specific taxonomic level of GM in our research was genus based on the existing genome wide association study, so the analyses at lower taxonomic levels (e.g., species and strains) is highly anticipated. Finally, many phenotypes have bidirectional causal effects, in which exposure and outcome influence each other. In our study, although the bidirectional causal effect has been estimated using two one-way MR Models, this approach may have overlooked the bidirectional feedback loop between the two phenotypes, leading to a possibility of bias in effect estimates [57].

## Conclusions

In summary, our innovative study reported a comprehensive exploration for the causal relationship between GM and AS through conducting MR analyses and provided a new exploration direction for the treatment of AS. *Lactobacillaceae family*, *Rikenellaceae family*, *Howardella genus*, *Actinobacteria class* and *Ruminococcaceae_NK4A214_group genus* are expected to become new therapeutic targets and monitoring indicators of therapeutic effect for AS. Inflammatory cytokines (IL23 and IFN-γ) played a potential mediating role from GM dysbiosis to AS, especially for *Actinobacteria class*. However, underlying mechanism about how these 5 GM taxa involved in the pathogenesis of AS, MR analyses of AS patients by gender and age, and further exploration of reverse causality are highly anticipated in the future.

## Supporting information

S1 File Detailed information on the IVs of 5 suggestive GM taxa. (PDF)

S2 File The final retained SNPs of 5 suggestive GM taxa and AS after directional harmonization. (PDF)

S3 File Detailed information on the IVs of AS. (PDF)

S4 File The final retained SNPs of AS and 5 suggestive GM taxa after directional harmonization. (PDF)

S5 File Causal relationship of GM on 7 inflammatory cytokines. (PDF)

## References

[1] Dougados M, Baeten D. Spondyloarthritis. Lancet. 2011; 377(9783): 2127–2137. doi: 10.1016/S0140-6736(11)60071-8 21684383

[2] Wen C, Zheng Z, Shao T, Liu L, Xie Z, Le Chatelier E, et al. Quantitative metagenomics reveals unique gut microbiome biomarkers in ankylosing spondylitis. Genome Biol. 2017; 18(1): 142. doi: 10.1186/s13059-017-1271-6 28750650 PMC5530561

[3] Dean LE, Jones GT, MacDonald AG, Downham C, Sturrock RD, Macfarlane GJ. Global prevalence of ankylosing spondylitis. Rheumatology (Oxford). 2014; 53(1): 650–657. doi: 10.1093/rheumatology/ket387 24324212

[4] Hwang MC, Ridley L, Reveille JD. Ankylosing spondylitis risk factors: a systematic literature review. Clin Rheumatol. 2021; 40(8): 3079–3093. doi: 10.1007/s10067-021-05679-7 33754220 PMC9044547

[5] Voruganti A, Bowness P. New developments in our understanding of ankylosing spondylitis pathogenesis. Immunology. 2020; 161(2): 94–102. doi: 10.1111/imm.13242 32696457 PMC7496782

[6] Braun J, van den Berg R, Baraliakos X, Boehm H, Burgos-Vargas R, Collantes-Estevez E, et al. 2010 update of the ASAS/EULAR recommendations for the management of ankylosing spondylitis. Annals of the Rheumatic Diseases, 2011; 70(6): 896–904. doi: 10.1136/ard.2011.151027 21540199 PMC3086052

[7] Klingberg E, Magnusson MK, Strid H, Deminger A, Ståhl A, Sundin J, et al. A distinct gut microbiota composition in patients with ankylosing spondylitis is associated with increased levels of fecal calprotectin. Arthritis Res Ther. 2019; 21(1): 248. doi: 10.1186/s13075-019-2018-4 31771630 PMC6880506

[8] Adak A, Khan MR. An insight into gut microbiota and its functionalities. Cell Mol Life Sci. 2019; 76(3): 473–493. doi: 10.1007/s00018-018-2943-4 30317530 PMC11105460

[9] Song ZY, Yuan D, Zhang SX. Role of the microbiome and its metabolites in ankylosing spondylitis. Front Immunol. 2022; 13: 1010572. doi: 10.3389/fimmu.2022.1010572 36311749 PMC9608452

[10] Lee M, Chang EB. Inflammatory Bowel Diseases (IBD) and the Microbiome-Searching the Crime Scene for Clues. Gastroenterology. 2021; 160(2): 524–537. doi: 10.1053/j.gastro.2020.09.056 33253681 PMC8098834

[11] Alpizar-Rodriguez D, Lesker TR, Gronow A, Gilbert B, Raemy E, Lamacchia C, et al. Prevotella copri in individuals at risk for rheumatoid arthritis. Ann Rheum Dis. 2019; 78(5): 590–593. doi: 10.1136/annrheumdis-2018-214514 30760471

[12] Zhou C, Zhao H, Xiao XY, Chen BD, Guo RJ, Wang Q, et al. Metagenomic profiling of the pro-inflammatory gut microbiota in ankylosing spondylitis. J Autoimmun. 2020; 107: 102360. doi: 10.1016/j.jaut.2019.102360 31806420

[13] Liu B, Ding Z, Xiong J, Heng X, Wang H, Chu W. Gut Microbiota and Inflammatory Cytokine Changes in Patients with Ankylosing Spondylitis. Biomed Res Int. 2022; 2022: 1005111. doi: 10.1155/2022/1005111 36033581 PMC9417757

[14] Huang R, Li F, Zhou Y, Zeng Z, He X, Fang L, et al. Metagenome-wide association study of the alterations in the intestinal microbiome composition of ankylosing spondylitis patients and the effect of traditional and herbal treatment. J Med Microbiol. 2020; 69 (6): 797–805. doi: 10.1099/jmm.0.001107 31778109 PMC7451032

[15] Fan J, Zhou Y, Meng R, Tang J, Zhu J, Aldrich MC, et al. Cross-talks between gut microbiota and tobacco smoking: a two-sample Mendelian randomization study. BMC Med. 2023; 21(1): 163. doi: 10.1186/s12916-023-02863-1 37118782 PMC10148467

[16] Sekula P, Del Greco M F, Pattaro C, Köttgen A. Mendelian Randomization as an Approach to Assess Causality Using Observational Data. J Am Soc Nephrol. 2016, 27(11): 3253–3265. doi: 10.1681/ASN.2016010098 27486138 PMC5084898

[17] Davies NM, Holmes MV, Davey Smith G. Reading Mendelian randomisation studies: a guide, glossary, and checklist for clinicians. BMJ. 2018; 362: k601. doi: 10.1136/bmj.k601 30002074 PMC6041728

[18] Liu Y, Zhang G, Guan Y, Zhao X, Wang Q, Li H, et al. Association of IFN-γ polymorphisms with ankylosing spondylitis risk. J Cell Mol Med. 2020; 24(18): 10615–10620. doi: 10.1111/jcmm.15680 . 32729668 PMC7521230

[19] Xu H, Li B. Effect of Interferon-γ Polymorphisms on Ankylosing Spondylitis: A Case-Control Study. Med Sci Monit. 2017; 23: 4126–4131. doi: 10.12659/msm.902822 . 28843049 PMC5584821

[20] Xia Y, Liang Y, Guo S, Yu JG, Tang MS, Xu PH, et al. Association between cytokine gene polymorphisms and ankylosing spondylitis susceptibility: a systematic review and meta-analysis. Postgrad Med J. 2018; 94(1115): 508–516. doi: 10.1136/postgradmedj-2018-135665 30322951

[21] Braga M, Lara-Armi FF, Neves JSF, Rocha-Loures MA, Terron-Monich MS, Bahls-Pinto LD, et al. Influence of IL10 (rs1800896) Polymorphism and TNF-α, IL-10, IL-17A, and IL-17F Serum Levels in Ankylosing Spondylitis. Front Immunol. 2021; 12: 653611. doi: 10.3389/fimmu.2021.653611 34290697 PMC8287882

[22] Ciccia F, Guggino G, Rizzo A, Saieva L, Peralta S, Giardina A, et al. Type 3 innate lymphoid cells producing IL-17 and IL-22 are expanded in the gut, in the peripheral blood, synovial fluid and bone marrow of patients with ankylosing spondylitis. Ann Rheum Dis. 2015; 74(9): 1739–47. doi: 10.1136/annrheumdis-2014-206323 25902790

[23] Toussirot É, Laheurte C, Gaugler B, Gabriel D, Saas P. Increased IL-22- and IL-17A-Producing Mucosal-Associated Invariant T Cells in the Peripheral Blood of Patients With Ankylosing Spondylitis. Front Immunol. 2018; 9: 1610. doi: 10.3389/fimmu.2018.01610 . 30057583 PMC6053500

[24] Chen B, Li J, He C, Li D, Tong W, Zou Y, et al. Role of HLA-B27 in the pathogenesis of ankylosing spondylitis (Review). Mol Med Rep. 2017; 15(4): 1943–1951. doi: 10.3892/mmr.2017.6248 28259985 PMC5364987

[25] Lee YH, Song GG. Associations between interleukin-23R polymorphisms and ankylosing spondylitis susceptibility: an updated meta-analysis. Z Rheumatol. 2019; 78(3): 272–280. doi: 10.1007/s00393-018-0472-z 29691688

[26] Du J, Sun J, Wen Z, Wu Z, Li Q, Xia Y, et al. Serum IL-6 and TNF-α Levels Are Correlated with Disease Severity in Patients with Ankylosing Spondylitis. Lab Med. 2022; 53(2): 149–155. doi: 10.1093/labmed/lmab029 34415341

[27] Gratacós J, Collado A, Filella X, Sanmartí R, Cañete J, Llena J, et al. Serum cytokines (IL-6, TNF-alpha, IL-1 beta and IFN-gamma) in ankylosing spondylitis: a close correlation between serum IL-6 and disease activity and severity. Br J Rheumatol. 1994; 33(10): 927–31. doi: 10.1093/rheumatology/33.10.927 7921752

[28] Hu N, Chen X, Wang S, Yuan G, Wang Q, Shu H, et al. The association of polymorphisms in TNF and ankylosing spondylitis in common population: a meta-analysis. Eur Spine J. 2021; 30(6): 1402–1410. doi: 10.1007/s00586-021-06845-w 33877454

[29] Sode J, Bank S, Vogel U, Andersen PS, Sørensen SB, Bojesen AB, et al. Genetically determined high activities of the TNF-alpha, IL23/IL17, and NFkB pathways were associated with increased risk of ankylosing spondylitis. BMC Med Genet. 2018; 19(1): 165. doi: 10.1186/s12881-018-0680-z 30208882 PMC6136164

[30] Kurilshikov A, Medina-Gomez C, Bacigalupe R, Radjabzadeh D, Wang J, Demirkan A, et al. Large-scale association analyses identify host factors influencing human gut microbiome composition. Nat Genet. 2021; 53 (2): 156–165. doi: 10.1038/s41588-020-00763-1 33462485 PMC8515199

[31] Sun BB, Maranville JC, Peters JE, Stacey D, Staley JR, Blackshaw J, et al. Genomic atlas of the human plasma proteome. Nature. 2018; 558(7708):73–79. doi: 10.1038/s41586-018-0175-2 . 29875488 PMC6697541

[32] Gilly A, Park YC, Png G, Barysenka A, Fischer I, Bjørnland T, et al. Whole-genome sequencing analysis of the cardiometabolic proteome. Nat Commun. 2020;11(1): 6336. doi: 10.1038/s41467-020-20079-2 . 33303764 PMC7729872

[33] Ahola-Olli AV, Würtz P, Havulinna AS, Aalto K, Pitkänen N, Lehtimäki T, et al. Genome-wide Association Study Identifies 27 Loci Influencing Concentrations of Circulating Cytokines and Growth Factors. Am J Hum Genet. 2017; 100(1): 40–50. doi: 10.1016/j.ajhg.2016.11.007 . 27989323 PMC5223028

[34] Burgess S, Butterworth A, Thompson SG. Mendelian randomization analysis with multiple genetic variants using summarized data. Genet Epidemiol. 2013, 37(7): 658–65. doi: 10.1002/gepi.21758 24114802 PMC4377079

[35] Jiang J, Shao M, Wu X. Vitamin D and risk of ankylosing spondylitis: A two-sample mendelian randomization study. Hum Immunol. 2022; 83 (1): 81–85. doi: 10.1016/j.humimm.2021.09.003 34521568

[36] Li H, Chen L, Yuan C, Yang H, Ma Z, Zuo J. Diet-derived antioxidants and osteoporosis: A Mendelian randomization study. PLoS One. 2023,18(11): e0293145. doi: 10.1371/journal.pone.0293145 38019728 PMC10686434

[37] Bowden J, Davey Smith G, Haycock PC, Burgess S. Consistent Estimation in Mendelian Randomization with Some Invalid Instruments Using a Weighted Median Estimator. Genet Epidemiol. 2016, 40(4): 304–14. doi: 10.1002/gepi.21965 27061298 PMC4849733

[38] Hartwig FP, Davey Smith G, Bowden J. Robust inference in summary data Mendelian randomization via the zero modal pleiotropy assumption. Int J Epidemiol. 2017, 46(6):1985–1998. doi: 10.1093/ije/dyx102 29040600 PMC5837715

[39] Milne RL, Kuchenbaecker KB, Michailidou K, Beesley J, Kar S, Lindstrom S, et al. Identification of ten variants associated with risk of estrogen-receptor-negative breast cancer. Nat Genet. 2017, 49(12): 1767–1778. doi: 10.1038/ng.3785 29058716 PMC5808456

[40] Burgess S, Foley CN, Allara E, Staley JR, Howson JMM. A robust and efficient method for Mendelian randomization with hundreds of genetic variants. Nat Commun. 2020, 11(1): 376. doi: 10.1038/s41467-019-14156-4 31953392 PMC6969055

[41] Alwali AY, Parkinson EI. Small molecule inducers of actinobacteria natural product biosynthesis. J Ind Microbiol Biotechnol. 2023; 50(1): kuad019. doi: 10.1093/jimb/kuad019 37587009 PMC10549211

[42] Dai Q, Xia X, He C, Huang Y, Chen Y, Wu Y, et al. Association of anti-TNF-α treatment with gut microbiota of patients with ankylosing spondylitis. Pharmacogenet Genomics. 2022; 32(7): 247–256. doi: 10.1097/FPC.0000000000000468 35852868 PMC9351697

[43] Asquith M, Davin S, Stauffer P, Michell C, Janowitz C, Lin P, et al. Intestinal Metabolites Are Profoundly Altered in the Context of HLA-B27 Expression and Functionally Modulate Disease in a Rat Model of Spondyloarthritis. Arthritis Rheumatol. 2017; 69(10): 1984–1995. doi: 10.1002/art.40183 28622455 PMC5623151

[44] Manasson J, Shen N, Garcia Ferrer HR, Ubeda C, Iraheta I, Heguy A, et al. Gut Microbiota Perturbations in Reactive Arthritis and Postinfectious Spondyloarthritis. Arthritis Rheumatol. 2018; 70(2):242–254. doi: 10.1002/art.40359 29073348 PMC5788722

[45] Gill T, Stauffer P, Asquith M, Laderas T, Martin TM, Davin S, et al. Axial spondyloarthritis patients have altered mucosal IgA response to oral and fecal microbiota. Front Immunol. 2022; 13: 965634. doi: 10.3389/fimmu.2022.965634 36248884 PMC9556278

[46] Huynh U, Zastrow ML. Metallobiology of Lactobacillaceae in the gut microbiome. J Inorg Biochem. 2023; 238: 112023. doi: 10.1016/j.jinorgbio.2022.112023 36270041 PMC9888405

[47] Costello ME, Ciccia F, Willner D, Warrington N, Robinson PC, Gardiner B, et al. Brief Report: Intestinal Dysbiosis in Ankylosing Spondylitis. Arthritis Rheumatol. 2015; 67(3): 686–691. doi: 10.1002/art.38967 25417597

[48] Dong L, Du H, Zhang M, Xu H, Pu X, Chen Q, et al. Anti-inflammatory effect of Rhein on ulcerative colitis via inhibiting PI3K/Akt/mTOR signaling pathway and regulating gut microbiota. Phytother Res. 2022; 36(5): 2081–2094. doi: 10.1002/ptr.7429 35229916

[49] Feng Y, Cui Y, Jin J, Huang S, Wei J, Yao M, et al. The Alterations of Gut Microbiome and Lipid Metabolism in Patients with Spinal Muscular Atrophy. Neurol Ther. 2023; 12(3): 961–976. doi: 10.1007/s40120-023-00477-6 37103747 PMC10134726

[50] Vanderpool C, Yan F, Polk DB. Mechanisms of probiotic action: Implications for therapeutic applications in inflammatory bowel diseases. Inflamm Bowel Dis. 2008; 14(11): 1585–1596. doi: 10.1002/ibd.20525 18623173

[51] Jenks K, Stebbings S, Burton J, Schultz M, Herbison P, Highton J. Probiotic therapy for the treatment of spondyloarthritis: a randomized controlled trial. J Rheumatol. 2010; 37(10): 2118–2125. doi: 10.3899/jrheum.100193 20716665

[52] Paine A, Ritchlin CT. Targeting the interleukin-23/17 axis in axial spondyloarthritis. Curr Opin Rheumatol. 2016; 28(4): 359–367. doi: 10.1097/BOR.0000000000000301 27152702 PMC5777167

[53] De Vos M, Mielants H, Cuvelier C, Elewaut A, Veys E. Long-term evolution of gut inflammation in patients with spondyloarthropathy. Gastroenterology. 1996; 110(6): 1696–1703. doi: 10.1053/gast.1996.v110.pm8964393 8964393

[54] Cui Z, Hou G, Meng X, Feng H, He B, Tian Y. Bidirectional Causal Associations Between Inflammatory Bowel Disease and Ankylosing Spondylitis: A Two-Sample Mendelian Randomization Analysis. Front Genet. 2020; 11: 587876. doi: 10.3389/fgene.2020.587876 33329731 PMC7710797

[55] Xu Q, Ni JJ, Han BX, Yan SS, Wei XT, Feng GJ, et al. Causal Relationship Between Gut Microbiota and Autoimmune Diseases: A Two-Sample Mendelian Randomization Study. Front Immunol. 2022; 12: 746998. doi: 10.3389/fimmu.2021.746998 35140703 PMC8819003

[56] Qian Q, Xu X, He H, Ji H, Zhang H, Ding Y, et al. Clinical patterns and characteristics of ankylosing spondylitis in China. Clin Rheumatol. 2017; 36(7): 15. doi: 10.1007/s10067-017-3660-3 28550390

[57] Zou J, Talluri R, Shete S. Approaches to estimate bidirectional causal effects using Mendelian randomization with application to body mass index and fasting glucose. PLoS One. 2024, 19(3): e0293510. doi: 10.1371/journal.pone.0293510 38457457 PMC10923437

