# Supplementary material for: Causal relationship between gut microbiota and ankylosing spondylitis and potential mediating role of inflammatory cytokines: A mendelian randomization study
Source: PLoS One. 2024 Jul 31;19(7):e0306792. doi: 10.1371/journal.pone.0306792 (PMC11290680; doi:10.1371/journal.pone.0306792)
Supplement: S1 File — (PDF) [file pone.0306792.s001.pdf]

S1 File Detailed information on the IVs of 5 suggestive GM taxa

Detailed information on the IVs of *Actinobacteria class*

chr.exposure	SNP	other_allele.exposure	effect_allele.exposure	beta.exposure	se.exposure	pval.exposure	F
2	rs182549	T	C	0.111	0.012	2.470E-20	85.377
1	rs6660520	G	A	0.071	0.013	1.220E-07	27.984
16	rs8047955	G	A	0.058	0.012	8.160E-07	24.320
13	rs7322849	C	T	0.094	0.019	9.870E-07	23.953
15	rs72767435	C	T	-0.126	0.027	3.880E-06	21.323
22	rs134366	A	G	0.112	0.024	1.930E-06	22.662
12	rs10841473	C	G	-0.058	0.012	2.220E-06	22.396
5	rs11745923	T	G	0.056	0.012	1.060E-06	23.812
10	rs1515761	C	T	0.076	0.017	7.210E-06	20.136
15	rs12899991	T	A	0.072	0.015	3.240E-06	21.668
1	rs12049045	G	A	0.051	0.011	8.010E-06	19.936
2	rs1376754	A	G	0.051	0.011	6.000E-06	20.489
17	rs11655079	C	T	-0.056	0.012	6.450E-06	20.351
5	rs6893855	A	C	-0.077	0.017	8.810E-06	19.754

8	rs8008304<br>0	G	T	0.156	0.035	7.590E-06	20.038
7	rs961091	A	G	0.050	0.011	8.440E-06	19.835
11	rs4945008	G	A	0.054	0.012	8.410E-06	19.842
6	rs857444	T	C	0.051	0.012	1.010E-05	19.500

Detailed information on the IVs of *Lactobacillaceae family*

chr.exposure	SNP	other_allele.exposure	effect_allele.exposure	beta.exposure	se.exposure	pval.exposure	F
1	rs16861661	A	G	-0.193	0.038	3.470E-07	25.969
19	rs921925	C	A	0.100	0.020	7.220E-07	24.555
8	rs768253	G	T	-0.079	0.017	3.410E-06	21.570
20	rs6092149	T	A	-0.083	0.017	1.240E-06	23.510
16	rs328312	A	T	0.083	0.017	9.300E-07	24.068
2	rs11674854	T	C	-0.083	0.018	2.170E-06	22.438
3	rs77478751	G	A	-0.219	0.047	3.690E-06	21.421
1	rs74599091	G	A	0.192	0.043	6.540E-06	20.323
6	rs9345899	G	A	-0.124	0.028	8.720E-06	19.774
13	rs7399658	A	G	-0.105	0.022	2.070E-06	22.529
2	rs1530559	A	G	0.077	0.018	1.260E-05	19.063
4	rs62314653	A	C	0.177	0.039	6.350E-06	20.379

Detailed information on the IVs of *Rikenellaceae family*

chr.exposure	SNP	other_allele.exposure	effect_allele.exposure	beta.exposure	se.exposure	pval.exposure	F
13	rs9603208	T	G	0.082	0.016	2.670E-07	26.474
11	rs1939881	A	G	-0.106	0.021	3.050E-07	26.219
1	rs67705352	G	T	-0.055	0.011	6.590E-07	24.731
15	rs4264350	C	T	-0.053	0.011	1.240E-06	23.514
9	rs62532512	A	C	-0.050	0.011	2.660E-06	22.045
7	rs67281112	C	G	0.064	0.014	3.730E-06	21.397
4	rs6837275	G	A	0.057	0.012	1.600E-06	23.022
13	rs9578457	A	G	-0.141	0.032	7.490E-06	20.064
14	rs77885767	T	C	-0.156	0.034	3.440E-06	21.552
18	rs59663348	A	G	0.057	0.013	4.980E-06	20.845
21	rs8130320	G	A	-0.049	0.011	4.670E-06	20.968
14	rs74474130	G	T	0.138	0.030	3.180E-06	21.702
18	rs7242694	T	C	-0.062	0.013	4.500E-06	21.038
21	rs36021379	G	A	-0.066	0.014	5.980E-06	20.496
9	rs10217435	T	C	-0.088	0.020	7.660E-06	20.022
8	rs2447496	A	G	-0.055	0.012	6.630E-06	20.297
16	rs4783173	G	C	0.048	0.011	1.200E-05	19.160
8	rs7832304	G	T	-0.072	0.016	6.520E-06	20.330
11	rs10832801	C	A	-0.053	0.012	1.300E-05	19.005
6	rs9389714	T	C	-0.064	0.014	8.970E-06	19.718
2	rs6744030	T	C	0.070	0.016	9.210E-06	19.669

Detailed information on the IVs of *Howardella genus*

chr.exposure	SNP	other_allele.exposure	effect_allele.exposure	beta.exposure	se.exposure	pval.exposure	F
11	rs901099	G	T	-0.127	0.025	4.010E-07	25.689
18	rs1484873	G	A	-0.228	0.046	8.790E-07	24.177
7	rs17167098	A	G	-0.169	0.035	1.500E-06	23.142
4	rs609430	G	T	-0.112	0.024	2.850E-06	21.918
7	rs36081916	C	T	-0.181	0.040	6.890E-06	20.224
17	rs12452946	G	A	-0.106	0.023	3.790E-06	21.370
1	rs61771805	T	A	-0.137	0.030	4.060E-06	21.236
18	rs672217	A	G	0.164	0.035	2.730E-06	21.996
2	rs3791893	G	A	0.147	0.034	1.550E-05	18.677
15	rs10048062	T	C	-0.147	0.034	1.190E-05	19.172
14	rs2154047	A	C	-0.193	0.042	4.570E-06	21.010

Detailed information on the IVs of *Ruminococcaceae_NK4A214_group genus*

**chr.exposure**	**SNP**	other_allele.exposure	effect_allele.exposure	beta.exposure	se.exposure	pval.exposure	F
22	rs5994253	G	A	-0.081	0.016	2.640E-07	26.493
22	rs136761	A	G	-0.059	0.012	8.190E-07	24.312
1	rs11586410	A	G	-0.086	0.017	3.760E-07	25.815
12	rs34576931	C	G	-0.087	0.019	7.420E-06	20.081
2	rs114244418	G	C	-0.175	0.037	2.580E-06	22.105
20	rs4814689	T	C	-0.108	0.023	2.680E-06	22.036
7	rs73158814	G	C	-0.109	0.023	1.560E-06	23.073
2	rs12731	G	A	-0.053	0.012	4.510E-06	21.035

2	rs7573569	C	T	0.108	0.023	4.000E-06	21.264
3	rs147475196	G	A	-0.134	0.030	5.860E-06	20.535
16	rs62027366	C	T	0.062	0.014	7.750E-06	19.998
4	rs12642039	C	T	-0.055	0.012	3.630E-06	21.452
5	rs35559912	C	T	-0.093	0.020	5.570E-06	20.629
3	rs13087692	G	T	0.057	0.013	5.050E-06	20.818
1	rs6681678	T	C	-0.100	0.024	2.990E-05	17.422
5	rs11241747	T	C	0.053	0.012	8.690E-06	19.781
