# Supplementary material for: Causal relationship between gut microbiota and ankylosing spondylitis and potential mediating role of inflammatory cytokines: A mendelian randomization study
Source: PLoS One. 2024 Jul 31;19(7):e0306792. doi: 10.1371/journal.pone.0306792 (PMC11290680; doi:10.1371/journal.pone.0306792)
Supplement: S2 File — (PDF) [file pone.0306792.s002.pdf]

S2 File The final retained SNPs of 5 suggestive GM taxa and AS after directional harmonization

Retained SNPs of *Actinobacteria class* and AS after directional harmonization

SNP	effect_allele.exposure	other_allele.exposure	effect_allele.outcome	other_allele.outcome	beta.exposure	beta.outcome	eaf.outcome	se.outcome	pval.outcome	se.exposure	pval.exposure
rs10841473	G	C	G	C	-0.058	0.022	0.231	0.044	0.623	0.012	2.218E-06
rs11655079	T	C	T	C	-0.056	-0.067	0.187	0.048	0.163	0.012	6.447E-06
rs11745923	G	T	G	T	0.056	0.071	0.394	0.038	0.063	0.012	1.062E-06
rs12049045	A	G	A	G	0.051	0.070	0.383	0.038	0.067	0.011	8.010E-06
rs12899991	A	T	A	T	0.072	-0.005	0.244	0.044	0.909	0.015	3.242E-06
rs134366	G	A	G	A	0.112	0.091	0.069	0.074	0.219	0.024	1.931E-06
rs1376754	G	A	G	A	0.051	0.031	0.493	0.037	0.401	0.011	5.998E-06
rs1515761	T	C	T	C	0.076	0.099	0.075	0.071	0.165	0.017	7.214E-06
rs182549	C	T	C	T	0.111	0.030	0.402	0.038	0.431	0.012	2.466E-20

rs4945008	A	G	A	G	0.054	0.040	0.391	0.038	0.301	0.012	8.411E-06
rs6660520	A	G	A	G	0.071	-0.065	0.271	0.042	0.120	0.013	1.223E-07
rs6893855	C	A	C	A	-0.077	-0.049	0.106	0.061	0.416	0.017	8.806E-06
rs72767435	T	C	T	C	-0.126	-0.065	0.055	0.082	0.426	0.027	3.880E-06
rs7322849	T	C	T	C	0.094	0.060	0.092	0.065	0.356	0.019	9.870E-07
rs80083040	T	G	T	G	0.156	0.144	0.044	0.091	0.114	0.035	7.594E-06
rs8047955	A	G	A	G	0.058	0.043	0.382	0.038	0.266	0.012	8.159E-07
rs857444	C	T	C	T	0.051	0.030	0.369	0.039	0.445	0.012	1.006E-05
rs961091	G	A	G	A	0.050	-0.008	0.372	0.039	0.836	0.011	8.443E-06

Retained SNPs of *Lactobacillaceae family* and AS after directional harmonization

SNP	effect_allele.exposure	other_allele.exposure	effect_allele.outcome	other_allele.outcome	beta.exposure	beta.outcome	eaf.outcome	se.outcome	pval.outcome	se.exposure	pval.exposure
rs1530559	G	A	G	A	0.077	-0.001	0.463	0.038	0.976	0.018	1.265E-05

rs16861661	G	A	G	A	-0.193	0.133	0.066	0.075	0.078	0.038	3.469E-07
rs328312	T	A	T	A	0.083	-0.004	0.461	0.037	0.913	0.017	9.301E-07
rs6092149	A	T	A	T	-0.083	-0.014	0.422	0.038	0.715	0.017	1.243E-06
rs62314653	C	A	C	A	0.177	-0.109	0.059	0.080	0.170	0.039	6.353E-06
rs7399658	G	A	G	A	-0.105	-0.017	0.184	0.049	0.735	0.022	2.070E-06
rs74599091	A	G	A	G	0.192	-0.099	0.019	0.139	0.477	0.043	6.540E-06
rs768253	T	G	T	G	-0.079	0.053	0.439	0.037	0.160	0.017	3.412E-06
rs77478751	A	G	A	G	-0.219	0.031	0.119	0.058	0.600	0.047	3.688E-06
rs921925	A	C	A	C	0.100	-0.026	0.214	0.045	0.568	0.020	7.222E-07
rs9345899	A	G	A	G	-0.124	0.097	0.105	0.061	0.109	0.028	8.716E-06

Retained SNPs of *Rikenellaceae family* and AS after directional harmonization

SNP	effect_allele.exposure	other_allele.exposure	effect_allele.outcome	other_allele.outcome	beta.exposure	beta.outcome	eaf.outcome	se.outcome	pval.outcome	se.exposure	pval.exposure

rs10217435	C	T	C	T	-0.088	0.064	0.139	0.054	0.234	0.020	7.657E-06
rs10832801	A	C	A	C	-0.053	0.028	0.284	0.042	0.507	0.012	1.304E-05
rs1939881	G	A	G	A	-0.106	0.048	0.059	0.080	0.550	0.021	3.049E-07
rs2447496	G	A	G	A	-0.055	0.065	0.268	0.042	0.127	0.012	6.631E-06
rs36021379	A	G	A	G	-0.066	0.044	0.159	0.051	0.387	0.014	5.977E-06
rs4264350	T	C	T	C	-0.053	0.050	0.459	0.037	0.182	0.011	1.240E-06
rs4783173	C	G	C	G	0.048	-0.052	0.392	0.038	0.171	0.011	1.202E-05
rs59663348	G	A	G	A	0.057	-0.006	0.247	0.043	0.897	0.013	4.981E-06
rs62532512	C	A	C	A	-0.050	-0.023	0.432	0.038	0.548	0.011	2.664E-06
rs6744030	C	T	C	T	0.070	0.004	0.214	0.046	0.930	0.016	9.209E-06
rs6837275	A	G	A	G	0.057	-0.028	0.301	0.041	0.500	0.012	1.601E-06
rs74474130	T	G	T	G	0.138	-0.153	0.036	0.102	0.132	0.030	3.185E-06
rs77885767	C	T	C	T	-0.156	0.087	0.046	0.089	0.326	0.034	3.443E-06

rs7832304	T	G	T	G	-0.072	-0.059	0.125	0.056	0.297	0.016	6.517E-06
rs8130320	A	G	A	G	-0.049	0.053	0.558	0.037	0.155	0.011	4.671E-06
rs9389714	C	T	C	T	-0.064	0.036	0.088	0.066	0.588	0.014	8.974E-06
rs9578457	G	A	G	A	-0.141	-0.042	0.048	0.087	0.628	0.032	7.488E-06
rs9603208	G	T	G	T	0.082	-0.063	0.101	0.062	0.311	0.016	2.670E-07

Retained SNPs of *Howardella genus* and AS after directional harmonization

SNP	effect_allele.exposure	other_allele.exposure	effect_allele.outcome	other_allele.outcome	beta.exposure	beta.outcome	eaf.outcome	se.outcome	pval.outcome	se.exposure	pval.exposure
rs10048062	C	T	C	T	-0.147	0.044	0.089	0.067	0.504	0.034	1.195E-05
rs12452946	A	G	A	G	-0.106	0.050	0.498	0.037	0.176	0.023	3.787E-06
rs1484873	A	G	A	G	-0.228	0.143	0.152	0.052	0.006	0.046	8.786E-07
rs17167098	G	A	G	A	-0.169	-0.022	0.137	0.054	0.692	0.035	1.505E-06
rs2154047	C	A	C	A	-0.193	0.028	0.088	0.065	0.671	0.042	4.569E-06

rs36081916	T	C	T	C	-0.181	-0.008	0.089	0.066	0.909	0.040	6.887E-06
rs3791893	A	G	A	G	0.147	-0.036	0.143	0.054	0.507	0.034	1.549E-05
rs609430	T	G	T	G	-0.112	-0.004	0.350	0.039	0.911	0.024	2.845E-06
rs61771805	A	T	A	T	-0.137	-0.006	0.117	0.058	0.912	0.030	4.060E-06
rs672217	G	A	G	A	0.164	-0.006	0.199	0.047	0.894	0.035	2.732E-06

Retained SNPs of *Ruminococcaceae_NK4A214_group genus* and AS after directional harmonization

SNP	effect_allele.exposure	other_allele.exposure	effect_allele.outcome	other_allele.outcome	beta.exposure	beta.outcome	eaf.outcome	se.outcome	pval.outcome	se.exposure	pval.exposure
rs11241747	C	T	C	T	0.053	-0.041	0.309	0.041	0.322	0.012	8.686E-06
rs114244418	C	G	C	G	-0.175	-0.026	0.040	0.096	0.786	0.037	2.581E-06
rs11586410	G	A	G	A	-0.086	-0.080	0.158	0.052	0.120	0.017	3.758E-07
rs12642039	T	C	T	C	-0.055	-0.073	0.363	0.039	0.059	0.012	3.628E-06
rs12731	A	G	A	G	-0.053	0.011	0.379	0.038	0.776	0.012	4.509E-06

rs13087692	T	G	T	G	0.057	-0.026	0.297	0.041	0.530	0.013	5.050E-06
rs136761	G	A	G	A	-0.059	0.013	0.362	0.039	0.734	0.012	8.192E-07
rs147475196	A	G	A	G	-0.134	-0.131	0.107	0.061	0.032	0.030	5.856E-06
rs34576931	G	C	G	C	-0.087	0.092	0.069	0.074	0.209	0.019	7.424E-06
rs35559912	T	C	T	C	-0.093	-0.062	0.121	0.057	0.276	0.020	5.574E-06
rs4814689	C	T	C	T	-0.108	-0.026	0.045	0.090	0.777	0.023	2.676E-06
rs5994253	A	G	A	G	-0.081	-0.111	0.143	0.053	0.037	0.016	2.644E-07
rs62027366	T	C	T	C	0.062	0.075	0.203	0.047	0.109	0.014	7.752E-06
rs6681678	C	T	C	T	-0.100	0.057	0.034	0.104	0.582	0.024	2.994E-05
rs73158814	C	G	C	G	-0.109	0.083	0.037	0.101	0.412	0.023	1.560E-06
rs7573569	T	C	T	C	0.108	0.071	0.061	0.077	0.355	0.023	4.001E-06
