# Supplementary material for: Causal relationship between gut microbiota and ankylosing spondylitis and potential mediating role of inflammatory cytokines: A mendelian randomization study
Source: PLoS One. 2024 Jul 31;19(7):e0306792. doi: 10.1371/journal.pone.0306792 (PMC11290680; doi:10.1371/journal.pone.0306792)
Supplement: S3 File — (PDF) [file pone.0306792.s003.pdf]

S3 File Detailed information on the IVs of AS

Detailed information on the IVs of AS

chr.exposure	pos.exposure	beta.exposure	se.exposure	p.exposure	SNP	effect_allele.exposure	other_allele.exposure	eaf.exposure	F
2	62581573	-0.221	0.039	9.670E-09	rs13033284	C	T	0.628	32.899
6	31224667	0.520	0.045	3.732E-31	rs9264277	C	T	0.730	134.622
6	31313339	1.943	0.059	1.000E-200	rs9265893	C	G	0.177	1091.471
6	24071028	0.821	0.092	3.823E-19	rs34982906	C	T	0.053	80.003
6	28332453	2.108	0.089	5.321E-125	rs16894011	A	T	0.073	565.806
6	31318585	2.635	0.070	1.000E-200	rs9391773	T	G	0.129	1408.815
6	25848911	0.374	0.056	1.897E-11	rs62394289	A	G	0.138	45.037
6	33901952	1.295	0.106	3.363E-34	rs79693223	T	C	0.045	148.762
6	30077135	-0.693	0.056	1.972E-35	rs9378220	A	C	0.227	154.374
6	30775277	0.360	0.047	1.432E-14	rs112733823	T	C	0.217	59.172

6	35741951	0.733	0.095	9.441E-15	rs76644067	A	G	0.047	60.005
7	5473610	0.985	0.100	1.017E-22	rs181316459	C	G	0.047	96.192
7	5340664	-0.563	0.081	4.098E-12	rs10807943	C	T	0.937	48.039
