# Supplementary material for: Causal relationship between gut microbiota and ankylosing spondylitis and potential mediating role of inflammatory cytokines: A mendelian randomization study
Source: PLoS One. 2024 Jul 31;19(7):e0306792. doi: 10.1371/journal.pone.0306792 (PMC11290680; doi:10.1371/journal.pone.0306792)
Supplement: S4 File — (PDF) [file pone.0306792.s004.pdf]

S4 File The final retained SNPs of AS and 5 suggestive GM taxa after directional harmonization

Retained SNPs of AS and *Actinobacteria class* after directional harmonization

SNP	effect_allele.exposure	other_allele.exposure	effect_allele.outcome	other_allele.outcome	beta.exposure	beta.outcome	eaf.exposure	se.outcome	pval.outcome	se.exposure	pval.exposure
rs10807943	C	T	C	T	-0.563	0.022	0.937	0.019	0.320	0.081	4.098E-12
rs13033284	C	T	C	T	-0.221	-0.007	0.628	0.011	0.516	0.039	9.670E-09
rs16894011	A	T	A	T	2.108	0.012	0.073	0.040	0.682	0.089	5.321E-125
rs62394289	A	G	A	G	0.374	-0.016	0.138	0.018	0.270	0.056	1.897E-11
rs76644067	A	G	A	G	0.733	0.023	0.047	0.038	0.651	0.095	9.441E-15
rs9378220	A	C	A	C	-0.693	-0.015	0.227	0.016	0.391	0.056	1.972E-35

Retained SNPs of AS and *Lactobacillaceae family* after directional harmonization

SNP	effect_allele.exposure	other_allele.exposure	effect_allele.outcome	other_allele.outcome	beta.exposure	beta.outcome	eaf.exposure	se.outcome	pval.outcome	se.exposure	pval.exposure
rs10807943	C	T	C	T	-0.563	0.059	0.937	0.030	0.052	0.081	4.098E-12
rs13033284	C	T	C	T	-0.221	0.005	0.628	0.017	0.794	0.039	9.670E-09
rs62394289	A	G	A	G	0.374	0.000	0.138	0.027	0.999	0.056	1.897E-11
rs9378220	A	C	A	C	-0.693	-0.062	0.227	0.025	0.017	0.056	1.972E-35

Retained SNPs of AS and *Rikenellaceae family* after directional harmonization

SNP	effect_allele.exposure	other_allele.exposure	effect_allele.outcome	other_allele.outcome	beta.exposure	beta.outcome	eaf.exposure	se.outcome	pval.outcome	se.exposure	pval.exposure
rs10807943	C	T	C	T	-0.563	-0.042	0.937	0.019	0.023	0.081	4.098E-12
rs13033284	C	T	C	T	-0.221	0.005	0.628	0.011	0.660	0.039	9.670E-09
rs16894011	A	T	A	T	2.108	-0.009	0.073	0.039	0.861	0.089	5.321E-125
rs62394289	A	G	A	G	0.374	-0.015	0.138	0.017	0.362	0.056	1.897E-11
rs76644067	A	G	A	G	0.733	0.019	0.047	0.037	0.627	0.095	9.441E-15

rs9378220	A	C	A	C	-0.693	-0.017	0.227	0.016	0.298	0.056	1.972E-35

Retained SNPs of AS and *Howardella genus* after directional harmonization

SNP	effect_allele.exposure	other_allele.exposure	effect_allele.outcome	other_allele.outcome	beta.exposure	beta.outcome	eaf.exposure	se.outcome	pval.outcome	se.exposure	pval.exposure
rs10807943	C	T	C	T	-0.563	0.001	0.937	0.041	0.970	0.081	4.098E-12
rs13033284	C	T	C	T	-0.221	-0.016	0.628	0.023	0.489	0.039	9.670E-09
rs62394289	A	G	A	G	0.374	0.041	0.138	0.036	0.286	0.056	1.897E-11
rs9378220	A	C	A	C	-0.693	-0.004	0.227	0.034	0.901	0.056	1.972E-35

Retained SNPs of AS and *Ruminococcaceae_NK4A214_group genus* after directional harmonization

SNP	effect_allele.exposure	other_allele.exposure	effect_allele.outcome	other_allele.outcome	beta.exposure	beta.outcome	eaf.exposure	se.outcome	pval.outcome	se.exposure	pval.exposure
rs10807943	C	T	C	T	-0.563	-2.066E-04	0.937	0.020	0.997	0.081	4.098E-12
rs13033284	C	T	C	T	-0.221	0.016	0.628	0.012	0.167	0.039	9.670E-09
rs16894011	A	T	A	T	2.108	0.012	0.073	0.043	0.749	0.089	5.321E-125

rs62394289	A	G	A	G	0.374	0.022	0.138	0.018	0.218	0.056	1.897E-11
rs76644067	A	G	A	G	0.733	-0.016	0.047	0.040	0.657	0.095	9.441E-15
rs9378220	A	C	A	C	-0.693	-0.005	0.227	0.017	0.854	0.056	1.972E-35
