# Supplementary material for: Causal relationship between gut microbiota and ankylosing spondylitis and potential mediating role of inflammatory cytokines: A mendelian randomization study
Source: PLoS One. 2024 Jul 31;19(7):e0306792. doi: 10.1371/journal.pone.0306792 (PMC11290680; doi:10.1371/journal.pone.0306792)
Supplement: S5 File — (PDF) [file pone.0306792.s005.pdf]

S5 File Causal relationship of GM on 7 inflammatory cytokines

Causal effects of *Actinobacteria class* on 7 inflammatory cytokines

Expoure	Outcome	Methods	N.SNPs	Beta	SE	P	$P\_{heterogeneity}$	$P\_{intercept}$
Actinobacteria class	IFN-γ	IVW	15	0.280	0.113	0.013	0.757	
		Weighted median		0.290	0.158	0.065		
		MR-Egger		0.357	0.331	0.300	0.693	0.809
		Weighted mode		0.314	0.221	0.177		
		Simple mode		0.376	0.274	0.192		
	IL-10	IVW	15	0.182	0.113	0.108	0.951	
		Weighted median		0.156	0.147	0.289		
		MR-Egger		0.394	0.331	0.255	0.943	0.508
		Weighted mode		0.098	0.221	0.664		
		Simple mode		0.272	0.259	0.310		

IL-17	IVW	15	0.110	0.072	0.127	0.704	
	Weighted median		0.050	0.109	0.644		
	MR-Egger		0.130	0.217	0.561	0.739	0.263
	Weighted mode		0.037	0.139	0.792		
	Simple mode		0.332	0.181	0.087		
IL-22	IVW	15	0.088	0.151	0.561	0.036	
	Weighted median		0.384	0.166	0.036		
	MR-Egger		0.432	0.446	0.361	0.034	0.439
	Weighted mode		0.355	0.200	0.098		
	Simple mode		0.266	0.282	0.362		
IL-23	IVW	15	0.339	0.113	0.003	0.552	
	Weighted median		0.151	0.166	0.363		
	MR-Egger		0.106	0.331	0.754	0.518	0.466
	Weighted mode		0.063	0.206	0.763		

	Simple mode		0.022	0.302	0.944		
IL-6	IVW	15	0.190	0.184	0.302	0.450	
	Weighted median		0.160	0.270	0.553		
	MR-Egger		0.343	0.526	0.526	0.461	0.298
	Weighted mode		0.267	0.399	0.513		
	Simple mode		0.505	0.448	0.277		
TNF-α	IVW	15	0.010	0.110	0.926	0.982	
	Weighted median		0.048	0.146	0.743		
	MR-Egger		0.013	0.336	0.971	0.969	0.944
	Weighted mode		0.085	0.189	0.658		
	Simple mode		0.109	0.235	0.649		

Causal effects of *Lactobacillaceae family* on 7 inflammatory cytokines

Expoure	Outcome	Methods	N.SNPs	Beta	SE	P	$P_{\_heterogeneity}$	$P_{\_intercept}$
Lactobacillaceae family	IFN-γ	IVW	10	0.139	0.092	0.133	0.725	
		Weighted median		0.190	0.120	0.115		
		MR-Egger		-0.111	0.256	0.675	0.753	0.324
		Weighted mode		0.228	0.197	0.277		
		Simple mode		0.244	0.188	0.227		
	IL-10	IVW	10	0.054	0.093	0.557	0.944	
		Weighted median		0.032	0.127	0.800		
		MR-Egger		-0.122	0.259	0.651	0.910	0.772
		Weighted mode		-0.030	0.199	0.882		
		Simple mode		-0.004	0.219	0.986		
	IL-17	IVW	10	0.165	0.071	0.019	0.251	
		Weighted median		0.067	0.092	0.464		

	MR-Egger		-0.041	0.191	0.837	0.284	0.280
	Weighted mode		0.049	0.130	0.714		
	Simple mode		0.063	0.170	0.719		
IL-22	IVW	10	0.074	0.093	0.421	0.636	
	Weighted median		0.146	0.121	0.227		
	MR-Egger		0.195	0.256	0.466	0.563	0.625
	Weighted mode		0.253	0.200	0.238		
	Simple mode		0.272	0.217	0.243		
IL-23	IVW	10	-0.032	0.140	0.820	0.014	
	Weighted median		-0.030	0.141	0.831		
	MR-Egger		-0.260	0.403	0.536	0.011	0.560
	Weighted mode		0.302	0.300	0.342		
	Simple mode		0.265	0.288	0.381		
IL-6	IVW	10	-0.130	0.171	0.447	0.731	

	Weighted median		-0.045	0.223	0.842		
	MR-Egger		-0.586	0.557	0.323	0.720	0.415
	Weighted mode		-0.035	0.316	0.915		
	Simple mode		-0.025	0.356	0.946		
TNF-α	IVW	10	-0.022	0.093	0.815	0.578	
	Weighted median		-0.008	0.130	0.951		
	MR-Egger		-0.397	0.256	0.159	0.748	0.154
	Weighted mode		0.003	0.183	0.989		
	Simple mode		-0.015	0.209	0.945		

Causal effects of *Rikenellaceae family* on 7 inflammatory cytokines

Expoure	Outcome	Methods	N.SNPs	Beta	SE	P	$P_{\_heterogeneity}$	$P_{\_intercept}$
Rikenellaceae family	IFN-γ	IVW	18	-0.033	0.115	0.777	0.353	
		Weighted median		-0.062	0.155	0.688		
		MR-Egger		0.161	0.353	0.654	0.314	0.568
		Weighted mode		-0.031	0.272	0.912		
		Simple mode		-0.031	0.287	0.916		
	IL-10	IVW	18	-0.054	0.129	0.677	0.143	
		Weighted median		-0.103	0.167	0.536		
		MR-Egger		0.764	0.337	0.037	0.417	0.020
		Weighted mode		-0.181	0.323	0.583		
		Simple mode		-0.248	0.319	0.446		
	IL-17	IVW	18	-0.010	0.075	0.183	0.664	
		Weighted median		-0.115	0.101	0.256		
		MR-Egger		0.364	0.250	0.164	0.852	0.070

	Weighted mode		-0.179	0.190	0.360		
	Simple mode		-0.163	0.181	0.378		
IL-22	IVW	18	0.027	0.110	0.810	0.844	
	Weighted median		0.153	0.149	0.304		
	MR-Egger		0.320	0.332	0.349	0.847	0.362
	Weighted mode		0.182	0.227	0.433		
	Simple mode		0.140	0.254	0.587		
IL-23	IVW	18	0.080	0.110	0.467	0.567	
	Weighted median		-0.019	0.157	0.902		
	MR-Egger		-0.156	0.332	0.645	0.538	0.461
	Weighted mode		-0.065	0.218	0.771		
	Simple mode		-0.035	0.246	0.889		
IL-6	IVW	18	0.100	0.169	0.553	0.773	
	Weighted median		0.172	0.238	0.469		
	MR-Egger		-0.199	0.483	0.686	0.745	0.518
	Weighted mode		0.107	0.353	0.765		

		Simple mode		0.215	0.399	0.597		
	TNF-α	IVW	18	0.670	0.125	0.593	0.208	
		Weighted median		0.089	0.161	0.581		
		MR-Egger		0.109	0.434	0.804	0.164	0.920
		Weighted mode		0.025	0.243	0.919		
		Simple mode		0.048	0.247	0.818		

Causal effects of *Howardella genus* on 7 inflammatory cytokines

Expoure	Outcome	Methods	N.SNPs	Beta	SE	P	$P\_{heterogeneity}$	$P\_{intercept}$
Howardella genus	IFN-γ	IVW	10	-0.041	0.073	0.576	0.631	
		Weighted median		-0.113	0.098	0.250		
		MR-Egger		0.244	0.327	0.478	0.617	0.399
		Weighted mode		-0.173	0.164	0.317		
		Simple mode		-0.171	0.161	0.318		
	IL-10	IVW	10	-0.038	0.079	0.632		

	Weighted median		-0.014	0.098	0.890		
	MR-Egger		0.256	0.358	0.495		0.425
	Weighted mode		-0.216	0.192	0.289		
	Simple mode		0.098	0.182	0.605		
IL-17	IVW	10	-0.020	0.045	0.664	0.433	
	Weighted median		-0.020	0.062	0.743		
	MR-Egger		-0.238	0.183	0.231	0.479	0.254
	Weighted mode		-0.017	0.097	0.863		
	Simple mode		-0.056	0.109	0.617		
IL-22	IVW	10	-0.111	0.073	0.128	0.464	
	Weighted median		-0.054	0.097	0.577		
	MR-Egger		-0.189	0.340	0.594	0.372	0.820
	Weighted mode		-0.018	0.144	0.905		
	Simple mode		-0.009	0.153	0.954		
IL-23	IVW	10	0.031	0.073	0.674	0.883	
	Weighted median		0.016	0.087	0.854		
	MR-Egger		0.227	0.327	0.507	0.856	0.555
	Weighted mode		-0.018	0.169	0.919		

	Simple mode		-0.024	0.152	0.880		
IL-6	IVW	10	-0.052	0.127	0.682	0.448	
	Weighted median		0.019	0.181	0.918		
	MR-Egger		-0.907	0.615	0.179	0.551	0.193
	Weighted mode		0.107	0.263	0.693		
	Simple mode		0.140	0.284	0.633		
TNF-α	IVW	10	-0.050	0.068	0.456	0.673	
	Weighted median		-0.046	0.090	0.606		
	MR-Egger		-0.512	0.274	0.098	0.889	0.120
	Weighted mode		0.027	0.144	0.858		
	Simple mode		0.062	0.159	0.704		

Causal effects of *Ruminococcaceae_NK4A214_group genus* on 7 inflammatory cytokines

Expoure	Outcome	Methods	N.SNPs	Beta	SE	P	$P\_{heterogeneity}$	$P\_{intercept}$
Ruminococcaceae_NK4A214_group genus	IFN-γ	IVW	13	0.205	0.129	0.113	0.411	
		Weighted median		0.153	0.176	0.385		
		MR-Egger		0.262	0.466	0.586	0.332	0.901
		Weighted mode		0.079	0.266	0.771		
		Simple mode		0.054	0.288	0.855		
	IL-10	IVW	13	0.014	0.127	0.914	0.651	
		Weighted median		0.050	0.174	0.774		
		MR-Egger		1.039	0.439	0.037	0.979	0.033
		Weighted mode		0.048	0.309	0.879		
		Simple mode		0.023	0.311	0.943	0.258	
	IL-17	IVW		0.043	0.095	0.650		
		Weighted median		0.163	0.120	0.175		

	MR-Egger		-0.078	0.339	0.821	0.205	0.715
	Weighted mode		0.192	0.191	0.334		
	Simple mode		0.183	0.210	0.400		
IL-22	IVW	13	-0.159	0.127	0.211	0.585	
	Weighted median		-0.033	0.169	0.847		
	MR-Egger		0.124	0.439	0.783	0.539	0.514
	Weighted mode		0.084	0.264	0.755		
	Simple mode		0.053	0.266	0.845		
IL-23	IVW	13	0.166	0.127	0.192	0.626	
	Weighted median		0.274	0.174	0.115		
	MR-Egger		-0.127	0.438	0.777	0.585	0.499
	Weighted mode		0.404	0.309	0.216		
	Simple mode		0.440	0.330	0.207		
IL-6	IVW	13	0.053	0.310	0.863	0.017	

	Weighted median		-0.439	0.317	0.166		
	MR-Egger		1.428	1.426	0.194	0.036	0.192
	Weighted mode		0.502	0.426	0.263		
	Simple mode		0.502	0.488	0.488		
TNF-α	IVW	13	0.041	0.129	0.750	0.630	
	Weighted median		0.140	0.162	0.388		
	MR-Egger		-0.719	0.445	0.135	0.825	0.103
	Weighted mode		0.148	0.270	0.592		
	Simple mode		0.148	0.266	0.587		
